# Isolation of Volatile Compounds by Microwave-Assisted Extraction from Six *Veronica* Species and Testing of Their Antiproliferative and Apoptotic Activities

**DOI:** 10.3390/plants12183244

**Published:** 2023-09-12

**Authors:** Ivana Vrca, Vedrana Čikeš Čulić, Mirela Lozić, Niko Dunkić, Dario Kremer, Mirko Ruščić, Marija Nazlić, Valerija Dunkić

**Affiliations:** 1 Faculty of Science, University of Split, Ruđera Boškovića 33, 21000 Split, Croatia; ivrca@pmfst.hr (I.V.); mrus@pmfst.hr (M.R.); mnazlic@pmfst.hr (M.N.); 2 School of Medicine, University of Split, Šoltanska 2, 21000 Split, Croatia; vcikesc@mefst.hr (V.Č.Č.); mirela.lozic@mefst.hr (M.L.); 3 Practice of General Medicine, Antuna Gustava Matoša 2, 21000 Split, Croatia; nikodunkic97@gmail.com; 4 Faculty of Pharmacy and Biochemistry, University of Zagreb, Ante Kovačića 1, 10000 Zagreb, Croatia; dkremer@pharma.hr

**Keywords:** *Veronica* species, microwave-assisted extraction, chemical composition, essential oil, hydrosol, antiproliferative activity, apoptotic activity

## Abstract

This study was conducted to determine the differences in the chemical composition of the essential oils and hydrosols of six different *Veronica* species (*V. agrestis*, *V. anagalloides*, *V. austriaca* ssp. *jacquinii*, *V. beccabunga*, *Veronica cymbalaria*, and *V. officinalis*) and to test their antiproliferative and apoptotic activities, according to the authors’ knowledge, because of insufficient research and lack of information. Also, the goal was to determine which obtained samples were better in achieving antiproliferative and apoptotic activities and due to which volatile components. Therefore, essential oils (EOs) and hydrosols (HYs) were isolated from the above-mentioned *Veronica* species by microwave-assisted extraction (MAE). Phytochemical identification of the free volatile compounds was performed using a GC equipped with a flame ionization detector and a mass spectrometer. Their antiproliferative and apoptotic activities against two human cancer cell lines, breast cancer cell line MDA-MB-231 and bladder cancer cell line T24, were determined. The main compounds identified in the studied *Veronica* EOs and HYs were terpinen-4-*ol* (0.34–6.49%), linalool (0.34–6.61%), (*E*)-caryophyllene (0.97–7.55%), *allo*-aromadendrene (0.18–2.21%), caryophyllene oxide (1.42–23.83%), benzene acetaldehyde (0.26–13.34%), and *β*-ionone (1.08–16.53%). In general, HYs of the tested *Veronica* species showed higher antiproliferative activity (IC_50_ 13.41–42.05%) compared to EOs (IC_50_ 158.1–970.4 µg/mL) on MDA-MB-231 and T24 cancer cell lines after 48 and 72 h. *V. agrestis* EO showed the best apoptotic effect among the EOs on the MDA-MB-231 cancer cell line (10.47 ± 0.53% and 9.06 ± 0.74% of early/late apoptosis, compared with control 3.61 ± 0.62% and 0.80 ± 0.17% of early/late apoptosis, respectively) and among the HYs *V. cymbalaria* showed 9.95 ± 1.05% and 3.06 ± 0.28% of early/late apoptosis and *V. anagalloides* 8.29 ± 1.09% and 1.95 ± 0.36% of early/late apoptosis compared with control (for EO was 7.45 ± 1.01% and 0.54 ± 0.25%, and for HY was 4.91 ± 1.97% and 0.70 ± 0.09% of early/late apoptosis, respectively) on the T24 cancer cell line. Future research will include other Croatian species of the genus *Veronica* to gain a more complete insight into the biological activity of the volatile products of this genus for potential discovery of drugs based on natural plant extracts.

## 1. Introduction

Throughout the long development of civilization, people have selected plants as food or/and medicine, primarily based on organoleptic evaluations. Scientific confirmation of the use of medicinal plants began with the development of analytical methods, especially chromatography. Chromatographic techniques enabled the identification and quantification of components of specialized metabolites from plant extracts and stimulated interest in studying the effects of these natural products on long-term health and preventive treatment [1]. Prior to the identification and quantification of interesting and potentially biologically active compounds, they must first be isolated from plant material. In their study, Dunkić et al. [2] reported the results of isolation of volatile compounds (VCs) by classical hydrodistillation and microwave-assisted extraction (MAE). Some VCs were only isolated with either hydrodistillation or MAE. The results obtained can be explained by the fact that hydrodistillation method is known to have some disadvantages, since the same compounds are decomposed because of the high temperatures and long extraction times. On the other hand, microwave distillation can sometimes lead to the isolation of few components, as stated in the study of Wu et al. [3]. However, modern techniques such as MAE are much faster, easier to use, and environmentally acceptable and enable extraction of bioactive components with less energy than conventional extraction methods [4,5]. Two types of samples can be obtained using the MAE technique, essential oil (EO), and hydrosol (HY). Essential oils are lipophilic, aromatized liquids with volatile constituents, obtained from plant material by steam distillation and named after the plant from which they originate [6]. Hydrosols are actually flavored waters obtained by condensation of water vapor in distillation processes [7] and contain a small quantity of water-soluble volatiles. Therefore, unlike EOs, HYs are safer for human use [8,9]. The general study of specialized plant metabolites contributes to the development of different areas of phytochemistry [10]. Specialized metabolites are very specific to certain plant families, genera, and species and contain an incredibly large library of bioactive compounds. Depending on the concentration, these compounds can also be toxic, and in adjusted doses, they represent a broad spectrum of phytochemical effects on human cells, bacteria, fungi, and parasites [10]. Genus *Veronica*, which has recently been studied because of its characteristic specialized metabolites, formerly a member of the Scrophulariaceae, was subsequently transferred to the Plantaginaceae family after phylogenetic and chemotaxonomic studies [11]. To date, research on specialized metabolites of the genus *Veronica* has mainly focused on iridoid glycosides, flavonoids, and saponins [12,13,14], while the free volatile compounds, which also constitute an important part of the specialized metabolites of this genus, have only recently begun to be studied in more detail [15,16,17]. Some *Veronica* species are used in traditional medicine worldwide for the treatment of different disorders such as the following: as an antiscorbutic, for wound healing, in respiratory diseases for cough, or as an expectorant [18]. Previous research on the *Veronica* species has shown that because of their specialized metabolites, they possess antioxidant, antimicrobial, cytotoxic, and antitumor activities [19,20,21]. Harput et al. [22] demonstrated the antiproliferative activity of investigated methanolic extracts from five *Veronica* species against two tumor cell lines, KB and B16 cells. Their results showed that the MeOH extracts possessed anti-inflammatory and cytotoxic activities. EOs have been demonstrated to possess anticancer properties through different mechanisms, including cancer prevention mechanisms, as well as the impact of the established tumor cell itself and the interaction with the microenvironment [23]. Key features of cancer include resistance to cell death. Therefore, therapeutic strategies are aimed to induce apoptosis [24]. In medical treatment, EOs and raw natural extracts are generally well accepted by patients, although their good reputation (because of the widespread belief that their naturalness is a guarantee of safety) may hide occasional toxicity problems because of the presence of specific components [25]. Despite different *Veronica* extracts having been used in traditional medicine for cancer treatment, only a few species have been studied for their cytotoxic and anticancer activity [14]. Apoptosis, programmed cell death, is a process that includes cell changes such as cell contraction, blebbing, DNA fragmentation, nuclear fragmentation, chromatin condensation, and mRNA decay [26]. Defects in apoptotic processes are associated with various diseases, including cancer. Uncontrolled cell proliferation is associated with insufficient apoptosis [26]. Therefore, scientific research is increasingly focused on medicinal plant research and the elucidation of signaling pathways that control cell cycle arrest and apoptosis. Because of all mentioned above, six species of the genus *Veronica* were selected for this study.

Consequently, the aim of this study was the phytochemical identification of FVCs in EOs and HYs isolated by microwave-assisted extraction (MAE) from six different *Veronica* species distributed in Croatia, *V. agrestis* L., *V. anagalloides* Guss., *V. austriaca* ssp. *jacquinii* L., *V. beccabunga* L., *V. cymbalaria* Bodard, and *V. officinalis* L., and determination of their antiproliferative and apoptotic activities against two human cancer cell lines: breast cancer cell line MDA-MB-231, and bladder cancer cell line T24. 

## 2. Results

### 2.1. Extraction of Volatile Components from Six Veronica Species

Extraction of volatiles from the six Croatian *Veronica* species collected in 2022 (Table 1) was performed by microwave-assisted extraction (MAE). Each extract consists of two parts, lipid and water, and both parts of the extracts of all studied species were analyzed by gas chromatography–mass spectrometry (GC-MS). The results of the composition of the lipid part (essential oil, EO) and the water part (hydrosol, HY) are presented in Table 2 and Table 3.

#### 2.1.1. Composition of Essential Oil 

The compounds linalool, (*E*)*-*caryophyllene, *allo-*aromadendrene, caryophyllene oxide, hexahydrofarnesyl acetone, phytol, *β*-ionone, hexadecanoic acid, docosane, tricosane, tetracosane, and octacosane were detected in six studied *Veronica* EOs (Table 2).

Peculiarities in the EO composition for each species were investigated. In the composition of *V. agrestis*, the dominant compound is phytol (56.57%); in *V. anagalloides*, the dominant compounds are hexahydrofarnesyl acetone (16.17%) and *β*-ionone (13.13%). In addition, in the composition of *V. austriaca* ssp. *jacquinii* EO, hexadecanoic acid (27.66%) and phytol (13.02%) are the predominant compounds. Phytol and hexadecanoic acid are also the predominant constituents in the species *V. beccabunga*, with 28.08% and 17.06%, respectively. In addition to these two compounds already mentioned, the compound caryophyllene oxide (23.83%) is significantly present in the species *V. cymbalaria*. Together with phytol and hexadecanoic acid, which are also significantly present in the EO composition of *V. officinalis*, heptacosane is the most abundant compound (17.21%) (Table 2).

#### 2.1.2. Composition of Hydrosols

The compounds terpinen-*4-ol*, linalool, (*E*)-caryophyllene, allo-aromadendrene, caryophyllene oxide, benzaldehyde, benzene acetaldehyde, and *β*-ionone were detected in the six studied *Veronica* HYs (Table 3).

In the composition of HY of *V. agrestis*, four components were identified with a proportion greater than 10%: caryophyllene oxide (14.01%), (*E*)*-β*-damascenone (12.42%), benzene acetaldehyde (11.56%), and *β*-ionone (10.32%). In *V. anagalloides* HY, *β*-ionone (16.53%), benzaldehyde (13.56%), methyl eugenol (13.57%), and (*E*)-*β*-damascenone (11.55%) dominate. In addition, methyl eugenol accounts for 37.01%, more than one-third of all identified components in the HY composition of *V. austriaca* ssp. *jacquinii*. The HY composition of *V. beccabunga* differs significantly from the other *Veronica* species studied, as the HY composition is dominated by *α*-pinene and piperitone with 17.11% and 19.54%, respectively. The HY of *V. cymbalaria* is rich in the phenolic constituents methyl eugenol (38.61%) and (*Z*)-methyl isoeugenol (31.32%). Methyl eugenol also predominates in the HY of *V. officinalis* with 22.01% and is the most abundant compound, followed by the compounds (*E*)-*β*-damascenone and *β*-ionone with an identification percentage of more than 14% (Table 3).

### 2.2. Cell Viability and Proliferation Using MTT Assay 

Cell viability and proliferation after treatment with essential oils (EOs) and hydrosols (HYs) of six *Veronica* species were determined and presented on two cancer cell lines, breast cancer cell line MDA-MB-231 and bladder cancer cell line T24, using MTT cell proliferation assay. The mentioned cancer cell lines were treated at concentrations of 50, 100, 250, 500, and 1000 µg/mL for EOs, while HYs were tested at different dilutions (10%, 20%, 30%, 40%, and 50%) after MAE for 4, 24, 48, and 72 h. After MTT cell proliferation assay, obtained results were expressed as % of metabolically active cells (Figure 1, Figure 2, Figure 3, Figure 4, Figure 5 and Figure 6 and Figures S1–S24) and IC_50_ values (50% cell growth inhibitory concentrations). In this work, only results for the times at which the samples showed antiproliferative activity in tested concentrations and dilutions are presented. In Supplementary Materials all the obtained results on MDA-MB-231 and T24 cancer cell lines are presented (Figures S1–S24).

**Figure 1:**
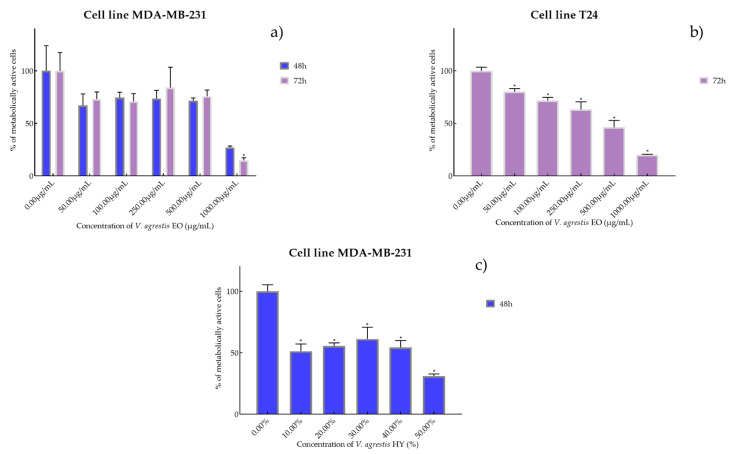
Metabolic activity of the cells (%) after treatment with *V. agrestis* EO (**a**,**b**) and *V. agrestis* HY (**c**) on MDA-MB-231 and T24 cancer cell lines. The results are expressed as means of three independent experiments with SD values (presented as error bars). For statistical analyses, a *t*-test with unequal variances was performed with the significance set at * *p* < 0.05.

Antiproliferative activity of *V. agrestis* EO on MDA-MB-231 after 48 and 72 h was IC_50_ 572.1 µg/mL and 586.9 µg/mL (percentage of metabolically active cells was 66.98%, 74.43%, 73.57% 71.43%, and 27.15% after 48 h, and 73.11%, 70.98%, 84.05%, 75.79%, and 14.86% after 72 h at concentrations of 50, 100, 250, 500, and 1000 µg/mL) and IC_50_ 340.6 µg/mL on T24 after 72 h (percentage of metabolically active cells was 80.18%, 71.73%, 63.31%, 46.32%, and 20.06% at concentrations of 50, 100, 250, 500, and 1000 µg/mL) (Figure 1a,b). On the other hand, *V. agrestis* HY showed only antiproliferative activity on the MDA-MB-231 cancer cell line after 48 h as IC_50_ 28.43%, and the percentage of metabolically active cells was 51.40%, 55.49%, 61.35%, 54.50%, and 31.11% at dilutions of 10%, 20%, 30%, 40%, and 50% (Figure 1c).

**Figure 2:**
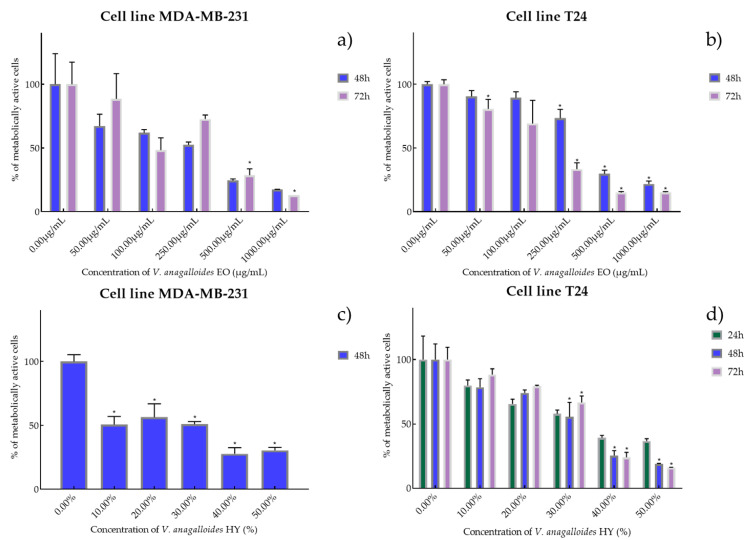
Metabolic activity of the cells (%) after treatment: with *V. anagalloides* EO (**a**,**b**) and *V. anagalloides* HY(**c**,**d**) on MDA-MB-231 and T24 cancer cell lines. The results are expressed as means of three independent experiments with SD values (presented as error bars). For statistical analyses, a *t*-test with unequal variances was performed with the significance set at * *p* < 0.05.

*V. anagalloides* EO showed antiproliferative activity after 48 and 72 h on the MDA-MB-231 cancer cell line with IC_50_ 180.1 µg/mL and 243.4 µg/mL (percentage of metabolically active cells was 67.19%, 61.90%, 52.41%, 24.43%, and 17.35% after 48 h, and 88.51%, 48.23%, 72.66%, 28.69%, 12.91% after 72 h at concentrations of 50, 100, 250, 500, and 1000 µg/mL) and T24 cancer cell line was IC_50_ 389.9 µg/mL and 158.1 µg/mL (percentage of metabolically active cells was 90.46%, 89.34%, 73.31%, 29.75%, and 21.83% after 48 h, and 80.67%, 69.20%, 33.35%, 15.01%, and 15.20% after 72 h at concentrations of 50, 100, 250, 500, and 1000 µg/mL) (Figure 2a,b). HY of *V. anagalloides* showed antiproliferative activity of 19.82% on MDA-MB-231 only after 48 h (percentage of metabolically active cells was 50.69%, 56.39%, 50.87% 27.69%, and 30.43% at dilutions of 10%, 20%, 30%, 40%, and 50%) and 33.70%, 26.96%, and 31.76% after 24, 48, and 72 h on T24 cancer cell lines (percentage of metabolically active cells was 79.91%, 65.66%, 57.93%, 39.38%, and 36.73% after 24 h, and 78.61%, 74.16%, 55.49%, 28.44%, and 18.85% after 48 h, and 88.45%, 79.50%, 66.72%, 24.10%, and 16.10% after 72 h at dilutions of 10%, 20%, 30%, 40%, and 50%) (Figure 2c,d).

**Figure 3:**
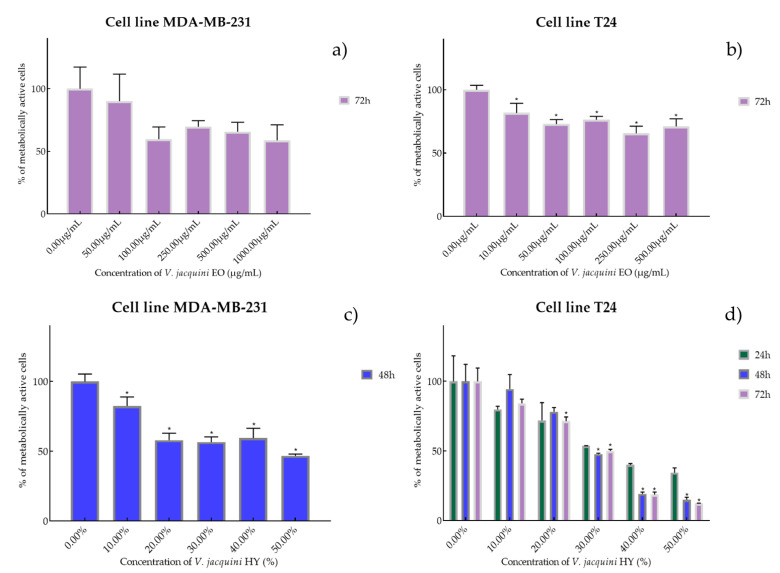
Metabolic activity of the cells (%) after treatment with *V. austriaca* ssp. *jacquini* EO (**a**,**b**) and *V. jacquini* HY (**c**,**d**) on MDA-MB-231 and T24 cancer cell lines. The results are expressed as means of three independent experiments with SD values (presented as error bars). For statistical analyses, a *t*-test with unequal variances was performed with the significance set at * *p* < 0.05.

Species *V. austriaca* ssp. *jacquinii* EO showed antiproliferative activity only after 72 h on both mentioned cancer cell lines as IC_50_ 816.0 µg/mL on MDA-MB-231 (percentage of metabolically active cells was 90.08%, 59.77%, 69.73%, 65.67%, and 58.83% after 72 h at concentrations of 50, 100, 250, 500, and 1000 µg/mL) and 617.1 µg/mL on T24 (percentage of metabolically active cells was 81.67%, 72.96%, 76.37%, 65.60%, and 71.11% after 72 h at concentrations of 50, 100, 250, 500, and 1000 µg/mL) (Figure 3a,b). In general, *V. austriaca* ssp. *jacquinii* HY showed better antiproliferative effect, especially on the T24 cancer cell line, than EO (percentage of metabolically active cells was 82.43%, 58.03%, 56.56%, 59.46%, and 46.73% after 48 h at dilutions of 10%, 20%, 30%, 40%, and 50%). After 48 h on MDA-MB-231, it showed an antiproliferative effect of 42.05% but pointed to the T24 cell line antiproliferative activity of 33.65%, 26.72%, and 23.58% (percentage of metabolically active cells was 79.82%, 71.84%, 53.61%, 40.01%, and 34.44% after 24 h, and 94.35%, 78.07%, 47.72%, 19.14%, and 14.91% after 48 h, and 84.10%, 71.34%, 50.04%, 18.61%, and 12.06% after 72 h at dilutions of 10%, 20%, 30%, 40%, and 50%) (Figure 3c,d). 

**Figure 4:**
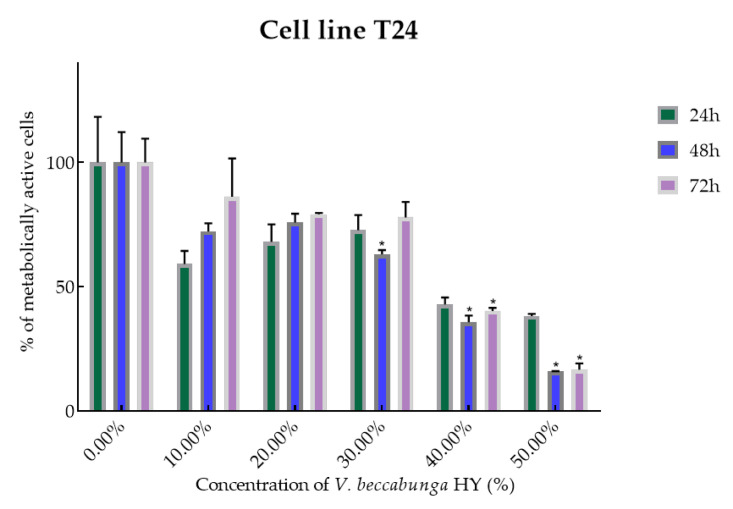
Metabolic activity of the cells (%) after treatment with *V. beccabunga* HY on the T24 cancer cell line. The results are expressed as means of three independent experiments with SD values (presented as error bars). For statistical analyses, a *t*-test with unequal variances was performed with the significance set at * *p* < 0.05.

*V. beccabunga* HY showed only antiproliferative effect on T24 cancer cell line, IC_50_ 36.05%, 29.64%, and 39.29% after 24, 48, and 72 h, and metabolically active cells are presented in Figure 4 (percentage of metabolically active cells was 59.06%, 67.99%, 72.69%, 42.76%, and 38.17% after 24 h, and 72.24%, 75.76%, 62.95%, 35.65%, and 15.78% after 48 h, and 86.11%, 79.07%, 77.84%, 40.19%, and 16.62% after 72 h at dilutions of 10%, 20%, 30%, 40%, and 50%). The EO isolated from *V. beccabunga* did not show any antiproliferative effect.

**Figure 5:**
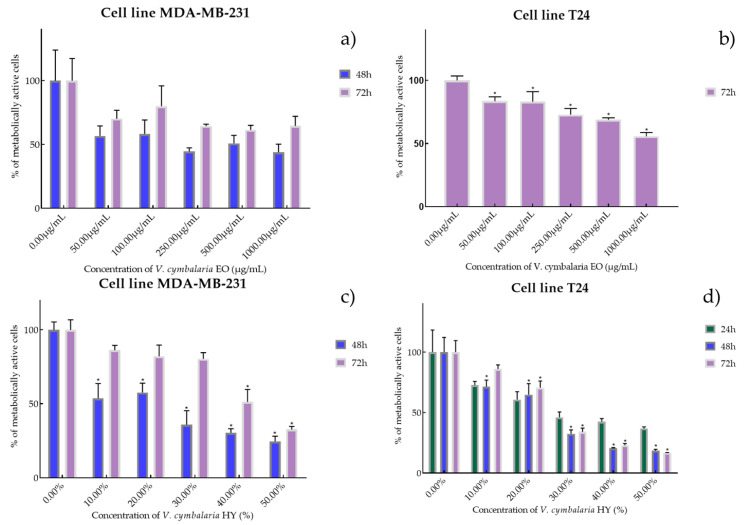
Metabolic activity of the cells (%) after treatment with *V. cymbalaria* EO (**a**,**b**) and *V. cymbalaria* HY (**c**,**d**) on MDA-MB-231 and T24 cancer cell lines. The results are expressed as means of three independent experiments with SD values (presented as error bars). For statistical analyses, a *t*-test with unequal variances was performed with the significance set at * *p* < 0.05.

EO of *V. cymbalaria* showed antiproliferative effect on the MDA-MB-231 cancer cell line (IC_50_ 249.9 µg/mL and 841.3 µg/mL after 48 and 72 h) (percentage of metabolically active cells was 56.72%, 58.29%, 44.57%, 50.92%, and 43.89% after 48 h, and 70.26%, 80.02%, 64.44%, 61.44%, and 64.63% after 72 h at concentrations of 50, 100, 250, 500, and 1000 µg/mL) compared to *V. cymbalaria* EO on the T24 cancer cell line (IC_50_ 970.4 µg/mL after 72 h) (percentage of metabolically active cells was 83.52%, 83.19%, 72.82%, 68.86%, and 55.73% after 72 h at concentrations of 50, 100, 250, 500, and 1000 µg/mL) (Figure 5a,b). HY of *V. cymbalaria* showed better antiproliferative effect than EO on MDA-MB-231 and T24 cancer cell lines as IC_50_ 17.41% and 49.63% on MDA-MB-231 after 48 and 72 h (percentage of metabolically active cells was 53.70%, 57.56%, 35.80%, 30.35%, and 24.51% after 48 h and 85.27%, 81.08%, 80.30%, 51.20%, and 31.54% after 72 h at dilutions of 10%, 20%, 30%, 40%, and 50% and IC_50_ 28.30%, 18.64%, and 22.28% on T24 after 24, 48 and 72 h, respectively) (percentage of metabolically active cells was 72.07%, 60.68%, 45.98%, 42.44%, and 36.68% after 24 h, and 71.44%, 64.76%, 32.34%, 20.62%, and 18.42% after 48 h, and 86.04%, 70.59%, 33.96%, 22.82%, and 16.76% after 72 h at dilutions of 10%, 20%, 30%, 40%, and 50%) (Figure 5c,d).

**Figure 6:**
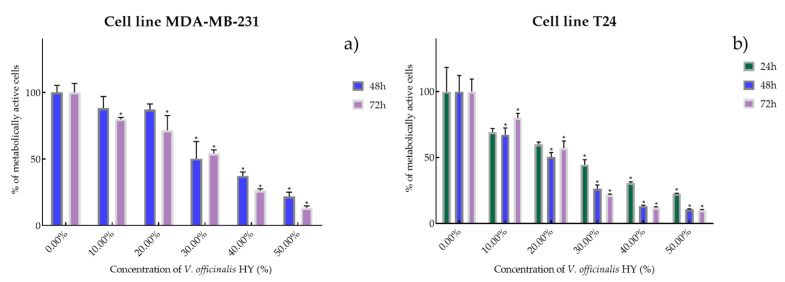
Metabolic activity of the cells (%) after *V. officinalis* HY on MDA-MB-231 (**a**) and T24 cancer cell lines (**b**). The results are expressed as means of three independent experiments with SD values (presented as error bars). For statistical analyses, a *t*-test with unequal variances was performed with the significance set at * *p* < 0.05.

It is interesting that the *V. officinalis* EO did not show antiproliferative activity at all four tested times (4, 24, 48, and 72 h), while *V. officinalis* HY showed excellent antiproliferative activity on MDA-MB-231 and T24 cancer cell lines. *V. officinalis* HY showed on MDA-MB-231 an antiproliferative effect of the cells after 48 and 72 h (IC_50_ 34.28% and 25.44%, respectively) (percentage of metabolically active cells was 88.22%, 87.17%, 50.25%, 37.40%, and 21.88% after 48 h, and 79.86%, 71.54%, 54.28%, 26.19%, and 13.43% after 72 h at dilutions of 10%, 20%, 30%, 40%, and 50%) and after 24, 48, and 72 h on the T24 cancer cell line as IC_50_ 21.83%, 13.41%, and 15.22% (percentage of metabolically active cells was 69.27%, 60.08%, 44.52%, 30.75%, and 22.55% after 24 h, and 67.25%, 50.52%, 26.40%, 13.23%, and 10.74% after 48 h, and 80.09%, 57.25%, 21.55%, 12.12%, and 10.04% after 72 h at dilutions of 10%, 20%, 30%, 40%, and 50%) (Figure 6a,b). 

### 2.3. Apoptotic Activity

**Figure 7:**
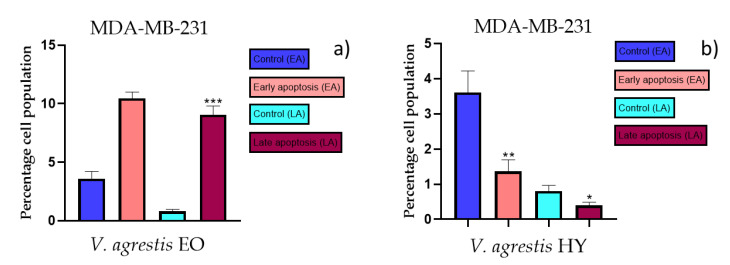
Apoptotic activity of *V. agrestis* EO and *V. agrestis* HY on the MDA-MB-231 cancer cell line (**a**,**b**). The percentages of apoptotic cells (Annexin-V-positive cells) were determined and presented as mean ± standard deviation (SD) with the significance set at * *p* < 0.05, ** *p* < 0.01, ***p < 0.001.

**Figure 8:**
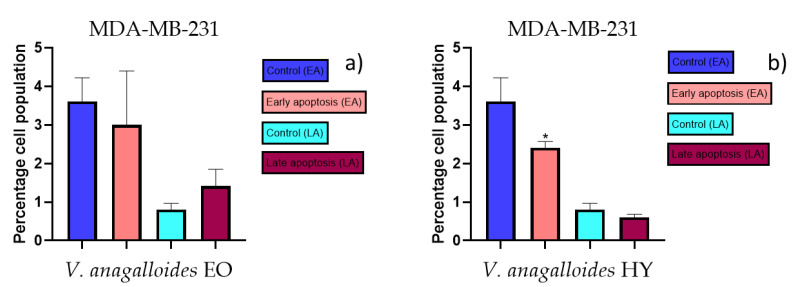
Apoptotic activity of *V. anagalloides* EO and *V. anagalloides* HY on the MDA-MB-231 cancer cell line (**a**,**b**). The percentages of apoptotic cells (Annexin-V-positive cells) were determined and presented as mean ± standard deviation (SD) with the significance set at * *p* < 0.05.

**Figure 9:**
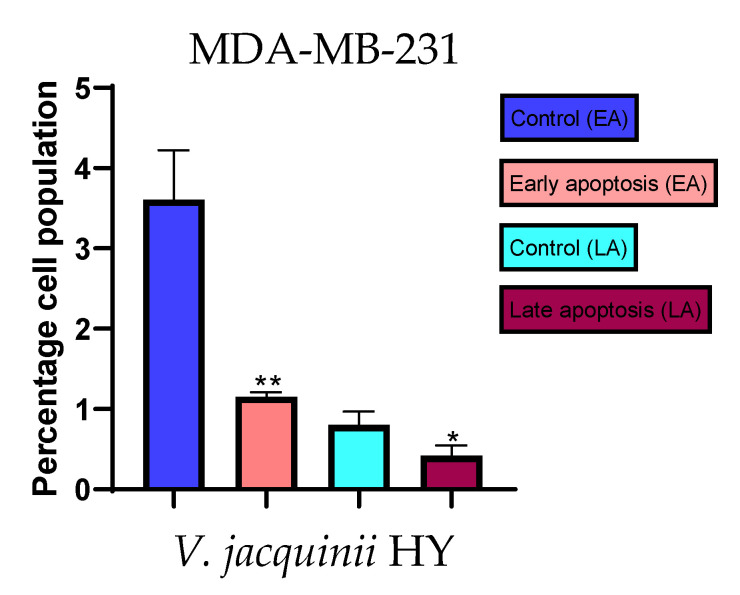
Apoptotic activity of *V. austriaca* ssp. *jacquinii* HY on the MDA-MB-231 cancer cell line. The percentages of apoptotic cells (Annexin-V-positive cells) were determined and presented as mean ± standard deviation (SD) with the significance set at * *p* < 0.05, ** *p* < 0.01.

**Figure 10:**
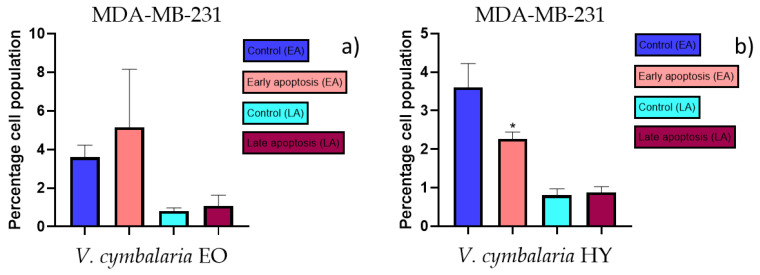
Apoptotic activity of *V. cymbalaria* EO and *V. cymbalaria* HY on the MDA-MB-231 cancer cell line (**a**,**b**). The percentages of apoptotic cells (Annexin-V-positive cells) were determined and presented as mean ± standard deviation (SD) with the significance set at * *p* < 0.05.

**Figure 11:**
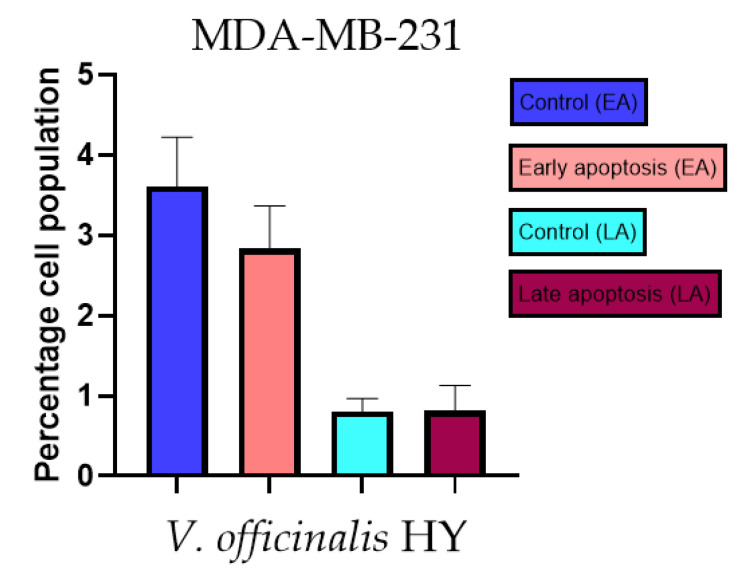
Apoptotic activity of *V. officinalis* HY on the MDA-MB-231 cancer cell line. The percentages of apoptotic cells (Annexin-V-positive cells) were determined and presented as mean ± standard deviation (SD).

The apoptotic activity of essential oils (EOs) and hydrosols (HYs) of some *Veronica* species that showed antiproliferative activity after 48 h was determined on two cancer cell lines: breast cancer cell line MDA-MB-231 and bladder cancer cell line T24. Results are expressed as the distinction between early (Annexin-V^+^/PI^−^) and late (Annexin-V^+^/PI^+^) apoptotic cells. All results are expressed as mean ± SD (*n* = 3). *V. agrestis* EO showed apoptotic activity on MDA-MB-231 of 10.47 ± 0.53% and 9.06 ± 0.74%, while *V. agrestis* HY showed 1.37 ± 0.33% and 0.40 ± 0.09% in early apoptosis and late apoptosis (Figure 7a,b), respectively. On the MDA-MB-231 cancer cell line, *V. anagalloides* EO showed apoptotic activity of 5.15 ± 2.99% and 1.07 ± 0.56%, while *V. anagalloides* HY showed on MDA-MB-231 2.41 ± 0.16 and 0.6 ± 0.08% in early apoptosis and late apoptosis (Figure 8a,b), respectively. *V. austriaca* ssp. *jacquinii* HY showed 1.15 ± 0.06% and 0.42 ± 0.13% in early apoptosis and late apoptosis, respectively, on the MDA-MB-231 cancer cell line (Figure 9). *V. cymbalaria* EO showed apoptotic activity on MDA-MB-231 of 5.15 ± 2.99% and 1.07 ± 0.56% in early apoptosis and late apoptosis, respectively (Figure 10a). HY of *V. cymbalaria* showed apoptotic activity of 2.26 ± 0.19% and 0.88 ± 0.15%, respectively (Figure 10b). *V. officinalis* HY showed 2.83 ± 0.53% and 0.81 ± 0.32% in early apoptosis and late apoptosis (Figure 11), respectively. Control on MDA-MB-231 showed 3.61 ± 0.62% and 0.80 ± 0.17% in early/late apoptosis, respectively.

**Figure 12:**
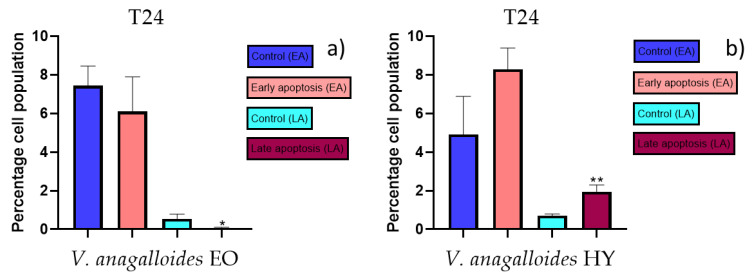
Apoptotic activity of *V. anagalloides* EO and *V. anagalloides* HY on the T24 cancer cell (**a**,**b**). The percentages of apoptotic cells (Annexin-V-positive cells) were determined and presented as mean ± standard deviation (SD) with the significance set at * *p* < 0.05, ** *p* < 0.01.

**Figure 13:**
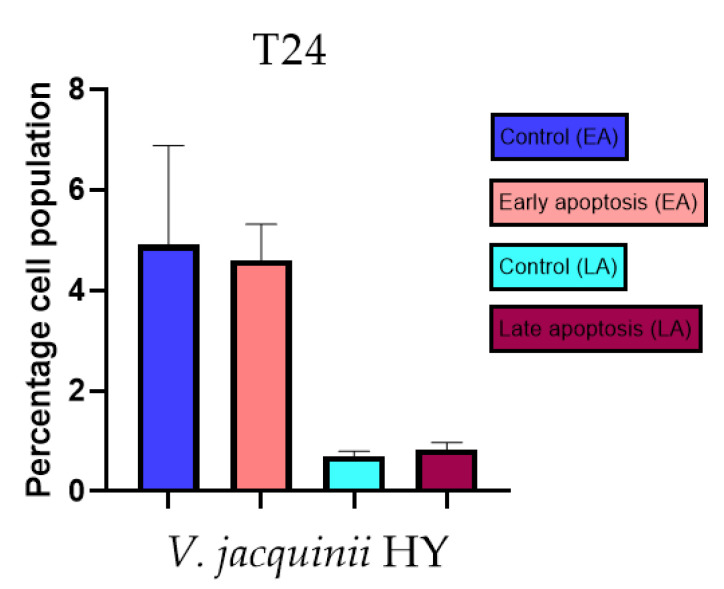
Apoptotic activity of *V. austriaca* ssp. *jacquinii* HY on the T24 cancer cell line. The percentages of apoptotic cells (Annexin-V-positive cells) were determined and presented as mean ± standard deviation (SD).

**Figure 14:**
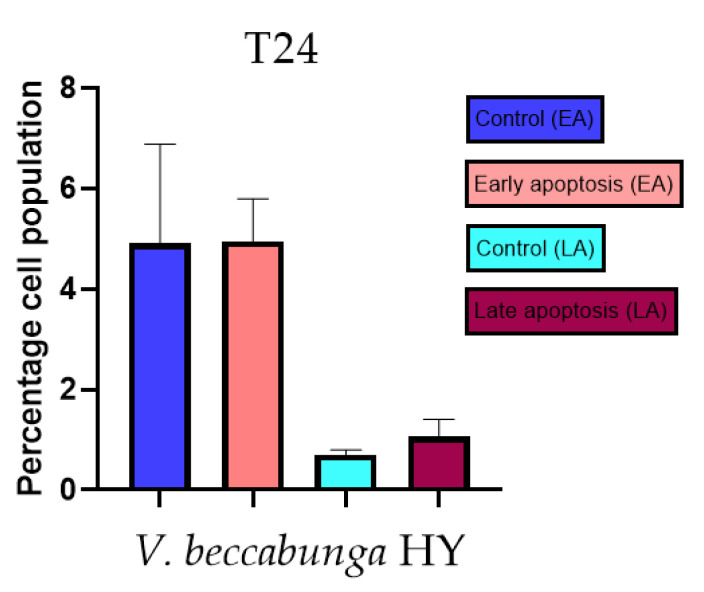
Apoptotic activity of *V. beccabunga* HY on the T24 cancer cell line. The percentages of apoptotic cells (Annexin-V-positive cells) were determined and presented as mean ± standard deviation (SD).

**Figure 15:**
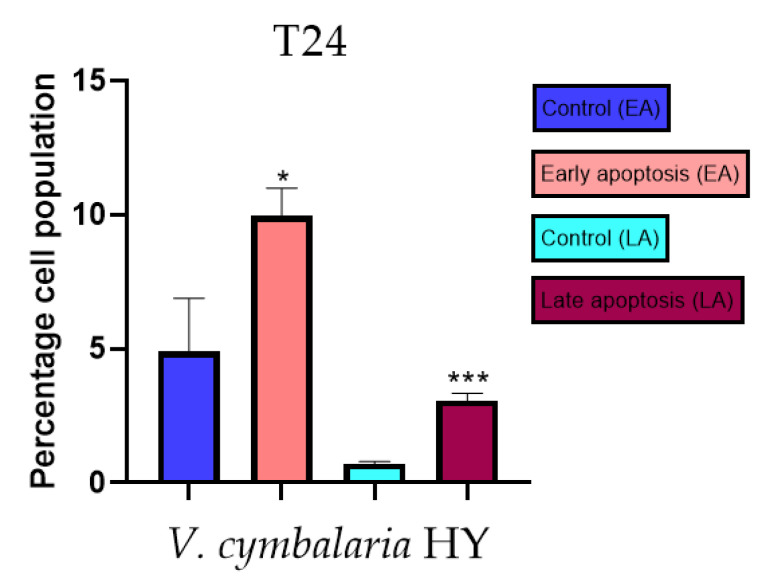
Apoptotic activity of *V. cymbalaria* HY on the T24 cancer cell line. The percentages of apoptotic cells (Annexin-V-positive cells) were determined and presented as mean ± standard deviation (SD) with the significance set at * *p* < 0.05, ***p < 0.001.

**Figure 16:**
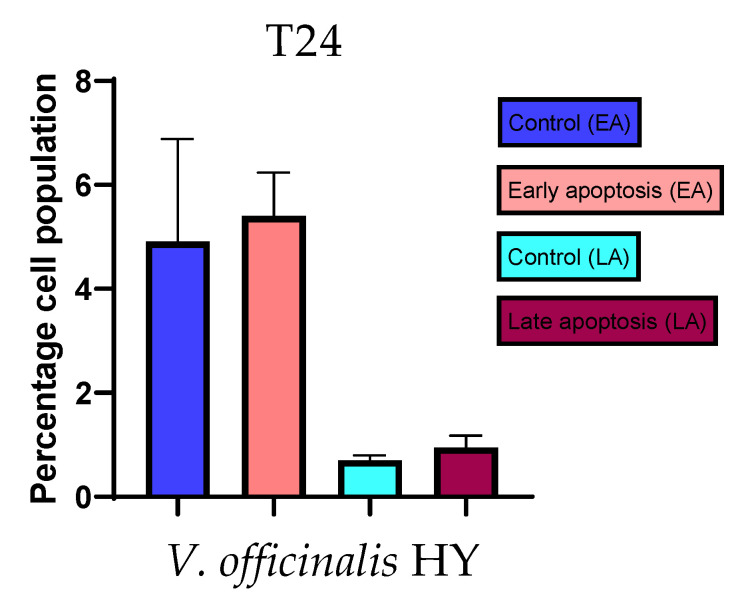
Apoptotic activity of *V. officinalis* HY on the T24 cancer cell line. The percentages of apoptotic cells (Annexin-V-positive cells) were determined and presented as mean ± standard deviation (SD).

Apoptotic activity of *V. anagalloides* EO was 6.1 ± 1.79% and 0.05 ± 0.0.5% (Figure 12a), while for *V. anagalloides*, HY was 8.29 ± 1.09% and 1.95 ± 0.36% (Figure 12b for early and late apoptosis on T24 bladder cancer cell line, respectively. Apoptotic activity of *V. austriaca* ssp. *jacquinii* HY on the T24 cancer cell line was 4.60 ± 0.72% for early apoptosis and 0.84 ± 0.13% for late apoptosis (Figure 13). Apoptotic activity of *V. beccabunga* HY on the T24 cancer cell line was 4.95 ± 0.85% for early apoptosis and 1.06 ± 0.35% for late apoptosis (Figure 14). HY of *V. cymbalaria* showed apoptotic activity on the T24 cancer cell line of 9.95 ± 1.05% and 3.06 ± 0.28%, respectively (Figure 15). *V. officinalis* HY showed apoptotic activity on the T24 cancer cell line of 5.41 ± 0.83% and 0.95 ± 0.22% for early and late apoptosis, respectively (Figure 16). Control for EO was 7.45 ± 1.01% and 0.54 ± 0.25%, and for HYs on the T24 cancer cell line was 4.91 ± 1.97% and 0.70 ± 0.09% in early apoptosis/late apoptosis, respectively.

## 3. Discussion

In this paper, the antiproliferative and apoptotic activities of free volatile compounds (FVCs) of six Croatian *Veronica* species, *V. agrestis*, *V. anagalloides*, *V. austriaca* ssp. *jacquinii*, *V. beccabunga*, *V. cymbalaria*, and *V. officinalis*, were investigated. The extraction of FVCs from all plant samples was performed by microwave-assisted extraction (MAE). Twelve samples were obtained—two samples for each species (essential oil (EO) and hydrosol (HY)). All samples were analyzed by gas chromatography–mass spectrometry (GC-MS), and the obtained data are presented in Table 2 and Table 3. The plant material of the mentioned *Veronica* species was collected in 2022 (Table 1).

In this study, FVCs isolated from the species *V. agrestis* were analyzed for the first time, and phytol was the dominant component in the fraction EO with 56.57% (Table 2). The fraction HY was dominated by caryophyllene oxide (14.01%), (*E*)*-β*-damascenone (12.42%), benzene acetaldehyde (11.56%), and *β*-ionone (10.32%) (Table 3). 

The FVCs of the other five *Veronica* species studied in this paper were compared with previously published data [2,7,28]. In the composition of the EO extract of *V. anagalloides*, the dominant compounds are hexahydrofarnesyl acetone (16.17%) and *β*-ionone (13.13%). Comparing the composition of the oil components of this species with the data previously published in the article by Dunkić et al. [2], we note a similarity in the concentration value of hexahydrofarnesyl acetone. In that research, the composition of oil components obtained by classical (Clevenger apparatus, HD) and modern hydrodistillation (MAE) was compared, so the value of hexahydrofarnesyl acetone in the EO of *V. anagalloides* was 14.33% for HD and 19.12% for MAE [2]. Moreover, in Nazlić et al. article [7], a much lower *β*-ionone relative percentage was found in this sample obtained by MAE compared to this study: only 4.22%.

In the composition of *V. austriaca* ssp. *jacquinii* EO, hexadecanoic acid (27.66%) and phytol (13.02%) were the predominant compounds (Table 2). Hexadecanoic acid was also the most abundant compound in a previously published study of *V. austriaca* ssp. *jacquinii* in MAE extract (22.17%) [2]. Phytol and hexadecanoic acid are also the predominant EO constituents in the species *V. beccabunga*, with 28.08% and 17.06%, respectively. The dominant compound in *V. beccabunga* collected in 2021 is oxygenated diterpene phytol with 34.54% for MAE [2], and the biggest difference in the composition of EO compounds in this line comparing 2021 and 2022 is the identification of piperitone: 29.28% in 2021 [2] and only 2.46% in this research.

Phytol was identified in the EO of *V. cymbalaria* with 3.71% in 2021 and 16.66% in 2022 (Table 2), while caryophyllene oxide with 23.83% was identified as less compared to in 2021 when 32.72% was identified [2]. Specifically, in the EO composition of *V. officinalis*, heptacosane is the most abundant compound (17.21%) (Table 2), while in the previous year, it was only identified as 5.52% [2].

The most significant differences in the proportions of the following compounds in the HY of *V. anagalloides* species are the following: in the extracts from 2022, the compound *β*-ionone was 16.53% and (*E*)*-β*-damascenone was 11.55% (Table 3), while in the material from 2021, these compounds were identified in significantly lower proportions, *β*-ionone with 6.07% and (*E*)*-β-*damascenone with 1.52% [7]. The reported differences in the EOs’ relative percentages of some compounds for the species, *V. anagalloides*, *V. austriaca* ssp. *jacquinii*, *V. cymbalaria*, and *V. officinalis*, could also be related to different locations of material collection, not just the difference in the year of collection.

Comparing the compositions of HYs from two years, the biggest discrepancy is the identification of methyl eugenol, which is 37.01% in the HY of *V. austriaca* ssp. *jacquinii* from 2022, while it was not identified at all in the year before; moreover, not a single phenolic component characteristic of species of the genus *Veronica* was identified [28]. The composition of *V. beccabunga* HY is dominated by α-pinene and piperitone at 17.11% and 19.54% (Table 3), respectively, and the differences compared with published compositions are as follows: piperitone was identified at 79.86% and α-pinene was not identified [28]. 

The HY of *V. cymbalaria* in this study is rich in the phenolic constituents methyl eugenol (38.61%) and (*Z*)-methyl isoeugenol (31.32%), while oxygenated sesquiterpene caryophyllene oxide is 6.26% (Table 3). Caryophyllene oxide is the most abundant compound in the previously published manuscript for the species *V. cymbalaria* HY with 37.12% [7]. The total phenolic components in *V. cymbalaria* in the research conducted on the material from 2021 are represented by only 5.51%, and the most abundant is thymol with 3.83%; methyl eugenol was not identified, and (*Z*)-methyl isoeugenol was identified with less than 1% [7]. Methyl eugenol is the most represented phenolic component in HY of *V. officinalis* with 22.01%, while in the previously published composition, the hydrosols of phenolic compounds was the only one represented by 2-methoxy-4-vinylphenol with 11.12%. The compounds (*E*)*-β*-damascenone and *β*-ionone for *V. officinalis* were identified in a similar percentage in both years compared (Table 3) [7]. From the comparison of the phytochemical profile of the *Veronica* species studied, it is evident that the composition of FVCs may vary within the same plant species, which is influenced by many factors such as abiotic and biotic factors, postharvest treatment, extraction methods, and storage conditions of the extract. Among the abiotic factors, microclimatic influence on plant growth is particularly important [29]. This is precisely why it is important to determine the chemical composition of any extract before beginning research into its biological activity. So far, almost 300 natural compounds from species of the genus *Veronica* have been identified and their biological activity studied [12,14], confirming the importance of this genus as medicinal plants.

Only a few studies have shown that extracts that contain terpenoids can behave synergistically with conventional chemotherapy. In spite of the encouraging results obtained over more than 35 years on the beneficial effects of these components, only a few clinical studies on humans have been conducted in the field. The only existing studies were conducted on limonene and its derivatives with some promising results [25].

So far, only a few *Veronica* species have been studied for their cytotoxic activity in vitro and in vivo, mostly methanolic and aqueous extracts of various *Veronica* species. Also, in our review, just two studies regarding antiproliferative activity of EOs and HYs were conducted [19,30]. In the present study, the antiproliferative activity of the EOs and HYs of the above-mentioned six *Veronica* species was tested on two cancer cell lines (MDA-MB-231 and T24). *V. agrestis* EO showed significant antiproliferative activity on MDA-MB-231 after 48 and 72 h (572.1 µg/mL and 586.9 µg/mL), and 340.6 µg/mL on T24 after 72 h. According to Nazlić et al. [30], similar activity was shown for *V. saturejoides* (Kamešnica Sample) on the HeLa, HCT116, and U2OS cell lines. EO of *V. anagalloides* showed the highest antiproliferative activity among all tested EOs (on the MDA-MB-231 cancer cell line, it was 180.1 µg/mL and 243.4 µg/mL after 48 and 72 h, respectively, and on the T24 cancer cell line, it was 389.9 µg/mL and 158.1 µg/mL after 48 and 72 h, respectively). Nazlić et al. [19] reported that *V. austriaca* ssp. *jacquinii* EO showed antiproliferative activity above 1000 µg/mL after 48 h on the tested cell lines, while in our present study, the EO of this species showed slightly better results, but after 72 h on both mentioned cancer cell lines (816.0 µg/mL on MDA-MB-231 and 617.1 µg/mL on T24). EO of *V. cymbalaria* showed an antiproliferative effect on the MDA-MB-231 cancer cell line (IC_50_ 249.9 µg/mL and 841.3 µg/mL after 48 and 72 h, respectively), while *V. cymbalaria* EO showed lower activity on the T24 cancer cell line (IC_50_ 970.4 µg/mL after 72 h). According to Nazlić et al. [30], *V. officinalis* EO showed significant antiproliferative activity on the HCT116 (IC_5O_ > 500 µg/mL), HeLa, and U2OS cancer cell lines (IC_5O_ > 1000 µg/mL). That could be a reason why the EOs of *V. officinalis* and *V. beccabunga* did not show antiproliferative activity in this study, as the intervals of tested concentrations were 50–1000 µg/mL. Generally, *V. agrestis* EO, *V. anagalloides* EO, and *V. cymbalaria* EO showed the highest antiproliferative activity of all tested *Veronica* species’ EOs. This could be due to the higher relative percentage of phytol, (*E*)-caryophyllene, and caryophyllene oxide in these species. For these compounds, significant antiproliferative and/or apoptotic activity was reported in recent studies [31,32,33].

HYs of *V. anagalloides*, *V. austriaca* ssp. *jacquinii*, and *V. cymbalaria* showed antiproliferative activity on MDA-MB-231 only after 48 h (17–43%). *V. beccabunga* HY did not show an antiproliferative effect at the tested dilutions and times on the MDA-MB-231 cancer cell line. HYs of *V. anagalloides*, *V. austriaca* ssp. *jacquinii*, *V. cymbalaria*, and *V. beccabunga* showed high antiproliferative activity (18–40%), especially on the T24 cancer cell line after 24, 48, and 72 h. In this study, HY of *V. officinalis* was the most effective in antiproliferative activity on the MDA-MB-231 (after 48 and 72 h, 34.28% and 25.44%, respectively) and T24 cell lines (after 24, 48, and 72 h on the T24 cancer cell line, 21.83% 13.41%, and 15. 22%, respectively). These results are in accordance with previous results on HYs of *V. officinalis* on HeLa, HCT116, and U2OS [30]. HY of *V. agrestis* did not show antiproliferative activity on the T24 cancer cell line. The reason for its biological inactivity potentially lies in the smaller amount of plant material from which the HY was obtained. The reason for the excellent antiproliferative activity of *V. officinalis* HY could be the presence of methyl eugenol, terpinen-4-ol, linalool, and *β*-ionone in higher relative percentages than in other tested HYs. For these compounds, significant antiproliferative and/or apoptotic activity was reported in recent studies [34,35,36,37,38,39]. Also, *β*-ionone was present in a higher percentage in *V. anagalloides* and *V. beccabunga* HYs, which could be the explanation for the great antiproliferative activity shown for these extracts, especially on the T24 cancer cell line. *V. beccabunga* HY also contained *α*-pinene in a high percentage, which could be explanation for the great antiproliferative activity on the T24 cancer cell line. There are many more studies of antiproliferative activity on various phenolic extracts of the *Veronica* species than on EOs and HYs. Harput et al. [22] reported that *V. hederifolia*, *V. cymbalaria*, *V. persica*, *V. pectinata var. glandulosa*, and *V. polita* methanolic extracts showed cytotoxic, anti-inflammatory, and radical-scavenging activities. *V. americana* methanolic extract showed antiproliferative activity on two cancer cell lines, HF-6 (colon cancer) and PC-3 (prostate cancer) [40]. According to Saracoglu and Harput [41], some iridoid glucosides of *V. persica* Poir, *V. anagallis*-*aquatica*, and *V. thymoides* subsp. *pseudocinerea* showed cytostatic and cytotoxic activities on human and murine cancer cell lines, with verminoside being the most cytotoxic component. Another study on *V. cuneifolia* subsp. *cuneifolia* and *V. cymbalaria* aqueous extracts showed moderate antiproliferative activity on Hep-2 (human epidermoid carcinoma), RD (human rhabdomyosarcoma), and L-20B (transgenic murine L-cells) [42]. 

Induction of apoptosis is extremely desirable for cancer control [43]. As far as the authors know, there are no previous studies of EOs and HYs from the *Veronica* species that have been tested for apoptotic activity. In general, EOs have been shown to induce intrinsic (mitochondria-dependent) and extrinsic (or cell death receptor-dependent) pathways of apoptosis. A study on the antitumor properties of caffeine isolated from the EO of *Piper cernuum* in melanoma cells showed that this component could induce apoptosis by activating the caspase-3 pathway, as well as by activating endoplasmic reticulum (ER) stress signaling. Another study focused on evaluating the mechanism of the action of carvacrol found in oregano and thyme EO. Carvacrol induced apoptosis in the MDA-MB-231 breast cancer cell line via mitochondrial membrane permeabilization resulted in the release of cytochrome C, and the induction of caspases was indicated by DNA cleavage and fragmentation [24]. According to Feng et al. [44], flavonoids extracted from *Veronica sibirica* (Vtfs) induced dose-dependent apoptosis in MCF-7 breast cancer cells with IC_50_ of 42 µg/mL. In our present study, apoptotic activity was tested on the previously mentioned *Veronica* species on MDA-MB-231 and the T24 cancer cell line. Mastelić et al. [45] reported paclitaxel in a concentration of 40 nM (36.24 µg/mL) for apoptotic activity on the MDA-MB-231 cancer cell line. According to Mastelić et al. [45], apoptotic activity of paclitaxel was 7.80% and for control, 1.58%, in apoptosis for the MDA-MB-231 cancer cell line. According to Bilušić et al. [46], cisplatin was used as a positive control against the T24 and A549 cancer cell lines for apoptotic activity in a concentration of 50 µg/mL. Cisplatin showed 1.36 ± 0.82% and 0.86 ± 0.14% in early/late apoptosis, respectively, on the T24 cancer cell line. In the present study, the best apoptotic activity of all tested EOs showed *V. agrestis* EO on the MDA-MB-231 cancer cell line (10.47 ± 0.53% of early apoptotic and 9.06 ± 0.74% of late apoptotic cells, comparing to control 3.61 ± 0.62% and 0.80 ± 0.17% of early/late apoptosis, respectively), and among the HYs, *V. cymbalaria* showed 9.95 ± 1.05% of early apoptotic and 3.06 ± 0.28% of late apoptotic cells, and *V. anagalloides* 8.29 ± 1.09% of early apoptotic and 1.95 ± 0.36% of late apoptotic cells, comparing to control (for EO was 7.45 ± 1.01% and 0.54 ± 0.25%, and for HYs was 4.91 ± 1.97% and 0.70 ± 0.09% of early/late apoptosis, respectively) on the T24 cancer cell line. To our knowledge, no other study involved testing apoptotic activity of the *Veronica* species’ EOs or HYs. 

The results obtained in this research showed comparison of EO and HY composition from the above-mentioned *Veronica* species, and significant antiproliferative and apoptotic activities of tested extracts and their possible chemotherapeutic properties, which is why they deserve further investigation. Also, one of the most extensive and interesting fields of application of nanobiotechnology is medicine using plant extracts. Ahmadov et al. [47] reported the process of green synthesis by *Scutellaria baicalensis* extract, and the components present in plant extract were the most important in the formation and stabilization of silver nanoparticles. These bioactive components can be connected to the surface of the silver nanoparticles and function as nanodrugs. Therefore, future research should be focused on FVCs present in the *Veronica* species on antiproliferative and apoptotic activities, their possible encapsulation for in vivo studies, and as potential nanodrugs in medicine.

## 4. Materials and Methods

### 4.1. Preparation, Extraction, and Identification of Volatile Compounds from Six Veronica Species

#### 4.1.1. Preparation of Plant Material from Six *Veronica* Species

All six *Veronica* species were collected during the flowering period in May and June 2022 at various locations in Croatia (Table 1). The voucher specimens were deposited in the herbarium of the Laboratory of Botany (HPMF-HR) of the Faculty of Science, University of Split, Croatia. All specimens were air dried in a single layer for 10 days and protected from direct sunlight.

#### 4.1.2. Extraction of Volatile Compounds

Plant material (30–50 g) of each *Veronica* species studied (Table 1) was hydrodistilled by microwave-assisted extraction (MAE) using an ETHOS X device (Milestone, Italy). MAE was performed at atmospheric pressure for 30 min (extraction process started after 10 min) at 800 W (98 °C). All extracts consisted of two layers: a lipophilic layer (essential oil) and a water layer (hydrosol). The lipophilic layer was collected in a side tube with a pentane/diethyl ether trap (VWR, Radnor, PA, USA), dried over anhydrous sodium sulfate, and stored at −20 °C until analysis. The pentane/diethyl ether trap was used because of the ease of evaporation (in order to exclude their toxicity), determining the yield of essential oil, and the certainty that there will be no loss of thermolabile components. The water extracts were also collected, and 2 g of HY from each sample was placed in a glass bottle and sealed with a stopper. The sample thus prepared was placed in a water bath, and a solid phase micro-extraction (SPME) needle was injected through the septum of the bottle cap. The first part of the process took place at 40 °C for 20 min to allow the compounds to evaporate from the water. The SPME fiber is directly above the liquid sample, which is stirred during the next 20 min of the process. The volatile compounds settled on the resin SPME fiber. The prepared sample was injected into the gas chromatography (GC) inlet and left there for 20 min to ensure that all volatile compounds were reabsorbed by the SPME fiber into the injection liner.

#### 4.1.3. Identification of Volatile Compounds

Chromatographic analyses were performed using a GC (model 3900; Varian Inc., Lake Forest, CA, USA) equipped with a flame ionization detector and a mass spectrometer (model 2100 T; Varian Inc., Lake Forest, CA, USA), a nonpolar capillary column VF-5 ms (30 m × 0.25 mm i.d., coating thickness 0.25 µm, Palo Alto, CA, USA), and a polar CP Wax 52 (30 m × 0.25 mm i.d., coating thickness 0.25 µm, Palo Alto, CA, USA). The chromatographic methods and conditions for hydrosol fraction analysis were the same as described in the article by Dunkić et al. [2]: the condition for the VF-5-ms column was a temperature of 60 °C (isothermal) for 3 min, which was then increased to 246 °C at a rate of 3 °C min^−1^ and maintained for 25 min (isothermal). The condition for the CP Wax 52 column was a temperature of 70 °C (isothermal) for 5 min, which was then increased to 240 °C at a rate of 3 °C min^−1^ and maintained for 25 min (isothermal). The injection volume was 2 µL, and the split ratio was 1:20. The MS conditions were as follows: ion source temperature, 200 °C; ionization voltage, 70 eV; mass scan range, 40–350 mass units. The individual peaks of all samples were identified by comparing their retention indices of n-alkanes with those of authentic samples and the studies [27,48] by comparison with our libraries from previous work and by comparison with other previously published material for the *Veronica* species [49,50,51]. Results are given as the mean of three analyses with standard deviation (*n* = 3 ± SD).

### 4.2. Cell Viability and Proliferation Were Determined by Measuring Cellular Metabolism Using MTT Assay

Cell viability and proliferation after treatment with essential oils (EOs) and hydrosols (HYs) of six different *Veronica* species (*V. agrestis*, *V. anagalloides*, *V. austriaca* ssp. *jacquinii*, *V. beccabunga, V. cymbalaria*, and *V. officinalis*) were performed against two human cancer cell lines, breast cancer cell line MDA-MB-231 and bladder cancer cell line T24, to determine which sample has the best antiproliferative activity. Stock solutions of EOs were prepared in dimethyl sulfoxide (DMSO) at a concentration of 10 mg/mL. Cell lines MDA-MB-231 and T24 were grown in Dulbecco’s modified Eagle’s medium (DMEM, Euroclone, Milano, Italy) in a humidified incubator at 37 °C with 5% CO_2_. The DMEM medium contained 10% fetal bovine serum (FBS, Euroclone, Milano, Italy) and 1% antibiotics (penicillin and streptomycin, Euroclone, Milano, Italy) and was used as a negative control. An equal number of cells (1 × 10^4^) were transferred into 96 wells and left overnight. The cells were then treated with EOs of the above-mentioned *Veronica* species at concentrations of 50, 100, 250, 500, and 1000 µg/mL, while HYs were tested at different dilutions (10%, 20%, 30%, 40%, and 50%) after MAE for 4, 24, 48, and 72 h. Cell viability and proliferation were determined by measuring cellular metabolism using an MTT assay. Yellow tetrazoline MTT (3-(4,5-dimethylthiazolid-2)-2,5-diphenyltetrazoline bromide) is reduced in metabolically active cells to the purple formazan. After 2 h, the medium with MTT was removed, and DMSO was added. The plates were incubated for 10 min at 37 °C with shaking. The absorbance was measured at 570 nm by a HiPo MPP-96 microplate photometer (Biosan, Riga, Latvia). All samples were run on three different plates in triplicate per plate. Solvent control was measured: the highest concentration of DMSO was adjusted to 1% (v/v) and did not show antiproliferative activity. According to Mastelić et al. [45], the antiproliferative activity of paclitaxel as positive control on MDA-MB-231 after 4, 24, 48, and 72 h in percentage of metabolically active cells was 82.04%, 83.85%, 49.21%, and 33.11%. According to Bilušić et al. [46], cisplatin was used as a positive control against T24 and A549 cancer cell lines for antiproliferative activity in a concentration of 0.05 mg/mL. Cisplatin showed antiproliferative activity after 4, 24, 48, and 72 h (percentages of metabolically active cells were 91.56%, 86.33%, 56.18%, and 52.32 for the T24 cancer cell line). The experiments and procedures are in accordance with ethical and safety guidelines. For statistical analyses, a *t*-test with unequal variances was performed using GraphPad Prism 9.0 statistical software (San Diego, CA, USA) with the significance set at * *p* < 0.05. The calculation of IC_50_ values was performed with GraphPad Prism software version 9.0 (San Diego, CA, USA), normalizing the data by three independent measurements of untreated controls.

### 4.3. Apoptotic Activity

An equal number of cells (1 × 10^4^) were seeded in 6-well plates and treated with essential oils (EOs) and hydrosols (HYs) of *V. agrestis*, *V. anagalloides*, *V. austriaca* ssp. *jacquinii*, *V. beccabunga*, *V. cymbalaria*, and *V. officinalis* for 48 h and then analyzed for apoptosis to determine which sample has the best apoptotic activity. The antiproliferative activities of *V. agrestis* EO and HY on the MDA-MB-231 cancer cell line after 48 were IC_50_ 572.1 µg/mL and 28.43%. *V. anagalloides* EO showed antiproliferative activity after 48 on the MDA-MB-231 and T24 cancer cell lines (IC_50_ 180.1 µg/mL and 389.9 µg/mL, respectively). HY of *V. anagalloides* showed antiproliferative activity of 19.82% on MDA-MB-231 and of 26.96% after 48 h on T24 cancer cell lines. *V. austriaca* ssp. *jacquinii* HY showed better antiproliferative effect, especially on the T24 cancer cell line. After 48 h on MDA-MB-231, it showed antiproliferative effect of 42.05%, but it pointed to the T24 cell line antiproliferative activity of 26.72% after 48 h. *V. austriaca* ssp. *jacquinii* HY showed on MDA-MB-231 an antiproliferative effect of 42.05% and pointed to the T24 cell line an antiproliferative activity of 26.72% after 48 h. *V. beccabunga* HY showed an antiproliferative effect on the T24 cancer cell line of 29.64% after 48 h. EO of *V. cymbalaria* also showed antiproliferative activity on the MDA-MB-231 cancer cell line (IC_50_ 249.9 µg/mL) after 48 h. HY of *V. cymbalaria* showed antiproliferative effect on the MDA-MB-231 and T24 cancer cell lines (17.41% and 18.64% after 48 h, respectively). *V. officinalis* HY showed on the MDA-MB-231 and T24 cancer cell lines significant antiproliferative effect after 48 h (34.28% and 13.41%, respectively). A combination of Annexin-V-FITC and propidium iodide staining allows the distinction between early (Annexin-V^+^/PI^−^) and late (Annexin-V^+^/PI^+^) apoptotic cells, necrotic cells, and live cells. After treatment with EOs and HYs, the cells were trypsinized, washed with PBS, and resuspended in 100 µL of the binding buffer containing 5 µL of Annexin-V-FITC and/or 10 µL of PI (FITC Annexin V Apoptosis Detection Kit with PI, BioLegend, San Diego, CA, USA). The cells were incubated for 15 min at room temperature in the dark and, thereafter, analyzed by flow cytometry (BD Accuri C6, BD Biosciences). Using the FlowLogic Software (Inivai, Mentone, VIC, Australia), the percentages of apoptotic cells (Annexin-V-positive cells) were determined and presented as mean ± standard deviation (SD). For statistical analyses of the apoptosis rate, a *t*-test with unequal variances, one-way ANOVA followed by a post hoc Tukey test or Kruskal–Wallis test, followed by Dunn’s post hoc test, were performed using GraphPad Prism 7.0 statistical software (San Diego, CA, USA), with the significance set at *p* < 0.05, *p* < 0.01 and *p* < 0.001.

## 5. Conclusions

Free volatile compounds (FVCs) present in essential oils (EOs) and hydrosols (HYs) of six *Veronica* species were obtained by microwave-assisted extraction (MAE). The identification of free volatile compounds (FVC) from six *Veronica* species was analyzed using gas chromatography–mass spectrometry, and the composition of the compounds was compared. The components identified in the EO extracts of all six *Veronica* species studied are linalool, (*E*)-caryophyllene, *allo*-aromadendrene, caryophyllene oxide, phytol, benzene acetaldehyde, *β*-ionone, hexadecanoic acid, docosane, tricosane, tetracosane, and octocosane. The constituents terpinen-4-ol, (*E*)-caryophyllene, *allo*-aromadendrene, caryophyllene oxide, benzaldehyde, benzene acetaldehyde, and β-ionone were identified in all studied HY extracts. These volatile compounds belong to the specialized metabolites, and it is important to know their composition for further research on the pharmaceutical activity of the *Veronica* species. Also, the antiproliferative and apoptotic effects of essential oils and hydrosols were compared. Generally, hydrosols showed better antiproliferative activity, especially on the T24 cancer cell line. Among the tested EOs, *V. anagalloides* exerted the best antiproliferative effect on both the MDA-MB-231 and T24 cancer cell lines, while among the tested HYs, *V. officinalis* HY showed the best antiproliferative activity. The best apoptotic effect of the EOs was shown by *V. agrestis* EO on the MDA-MB-231 cancer cell line, and among the HYs, *V. cymbalaria* and *V. anagalloides* showed the best proapoptotic activities on the T24 cancer cell line. Future research should be focused on the discovery of drugs based on natural plant extracts, which implies testing the combinatorial effect of compounds that show biological activity.

## Figures and Tables

**Table 1:** Details on collection data and origin of investigated *Veronica* species.

Species	Locality	Latitude	Longitude	Altitude a.s.l. (m)	Voucher No.
*V. agrestis*	Duba Stonska	42° 52′27.9″ N	17° 38′36.7″ E	12	CROVeS-22-2022
*V. anagalloides*	Njivice, Krk Island	45° 10′18.3″ N	14° 34′10.1″ E	6	CROVeS-07-2022
*V. austriaca* ssp. *jacquinii*	Korčula Island	42° 56′52.8″ N	16° 59′39.7″ E	441	CROVeS-02-2022
*V. beccabunga*	Podevčevo	46° 12′05.5″ N	16° 17′58.2″ E	215	CROVeS-08-2022
*V. cymbalaria*	Žrnovo, Korčula Island	42° 56′59.6″ N	17° 06′45.1″ E	153	CROVeS-03-2022
*V. officinalis*	Krasno, Velebit Mt	44° 49′52.4″ N	15° 01′06.9″ E	868	CROVeS-16-2022

**Table 2:** Constituents of the essential oils (EOs, %) obtained by microwave extraction of six *Veronica* species.

			*V. agrestis*	*V. anagalloides*	*V. austriaca* ssp. *jacquini*i	*V. beccabunga*	*V. cymbalaria*	*V. officinalis*
Component	RI^a^	RI^b^	EO ± SD	EO ± SD	EO ± SD	EO ± SD	EO ± SD	EO ± SD
Monoterpene hydrocarbons			1.38	1.17	0.88	-	-	-
*α*-Thujene	924	1012	0.65 ± 0.01	-	-	-	-	-
*α*-Pinene *	935	1017	0.73 ± 0.01	1.17 ± 0.01	0.88 ± 0.01	-	-	-
*β*-Phellandrene	1002	1195	-	-	-	-	-	-
Oxygenated monoterpenes			0.88	3.88	0.46	3.87	0.58	0.77
*γ*-Terpinene	1057	1225	-	0.33 ± 0.01	-	-	-	-
Linalool	1095	1506	0.34 ± 0.01	2.79 ± 0.01	0.46 ± 0.01	0.53 ± 0.01	0.58 ± 0.01	0.77 ± 0.01
Terpinen-4-*ol*	1174	1686	0.54 ± 0.01	0.76 ± 0.01	-	0.88 ± 0.01	-	-
*trans-p-*Mentha-1(7),8-dien-2-ol	1187	1803	-	-	-	-	-	-
Piperitone	1250	1719	-	-	-	2.46 ± 0.01	-	-
Sesquiterpene hydrocarbons			4.43	12.59	4.31	7.21	3.86	2.92
(*E*)*-*Caryophyllene *	1424	1585	3.25 ± 0.01	7.55 ± 0.01	3.08 ± 0.01	5.75 ± 0.01	2.53 ± 0.01	1.15 ± 0.01
*allo-*Aromadendrene	1465	1662	0.18 ± 0.03	1.21 ± 0.1	0.72 ± 0.05	0.81 ± 0.01	0.56 ± 0.01	1.77 ± 0.01
Germacrene D	1481	1692	0.41 ± 0.03	3.83 ± 0.01	0.81 ± 0.1	0.22 ± 0.01	0.77 ± 0.1	-
*δ-*Selinene	1492	1756	0.26 ± 0.01	-	-	0.43 ± 0.01	-	-
*δ*-Cadinene	1517	1745	0.33 ± 0.07	-	-	-	-	-
Oxygenated sesquiterpenes			7.58	25.43	18.64	12.08	34.9	16.07
Spathulenol	1577	2101	-	1.28 ± 0.05	-	-	-	-
Caryophyllene oxide *	1581	1955	2.11 ± 0.01	5.22 ± 0.01	7.35 ± 0.01	6.21 ± 0.01	23.83 ± 0.01	2.23 ± 0.01
Viridiflorol	1592	2099	-	-	-	0.45 ± 0.05	-	-
*γ*-Eudesmol	1632	2175	0.32 ± 0.01	0.56 ± 0.01	0.88 ± 0.01	0.73 ± 0.01	0.54 ± 0.01	-
*α*-Muurolol	1645	2181	-	-	-	-	-	3.93 ± 0.01
*α*-Bisabolol	1685	2210	-	1.43 ± 0.01	0.77 ± 0.01	-	-	0.32 ± 0.01
*α*-Bisabolol oxide	1748	2511	-	0.77 ± 0.01	-	-	-	0.75 ± 0.01
Hexahydrofarnesyl acetone *	1839	2113	5.15 ± 0.03	16.17 ± 0.01	9.64 ± 0.01	4.69 ± 0.01	10.53 ± 0.01	8.84 ± 0.01
Oxygenated diterpene			56.57	3.56	13.02	28.08	16.66	6.17
Phytol *	1942	2610	56.57 ± 0.01	3.56 ± 0.01	13.02 ± 0.01	28.08 ± 0.01	16.66 ± 0.01	6.17 ± 0.01
Phenolic compounds			0.77	2.02	-	-	1.38	5.06
Thymol *	1289	2154	0.77 ± 0.1	-	-	-	0.65 ± 0.01	-
Methyl eugenol	1403	2005	-	2.02 ± 0.01	-	-	0.73 ± 0.01	3.61 ± 0.01
(*Z*)-Methyl isoeugenol	1451	2070	-	-	-	-	-	1.45 ± 0.01
Acids, alcohols and esters			12.98	29.68	34.53	22.47	34.22	37.65
*3*-Hexen-1-ol	873	1383	-	-	-	-	-	0.46 ± 0.07
Benzaldehyde	952	1508	-	-	-	-	-	1.15 ± 0.01
Benzene acetaldehyde	1036	1633	0.26 ± 0.01	2.03 ± 0.01	0.86 ± 0.01	0.44 ± 0.01	8.61 ± 0.01	6.08 ± 0.01
*n-*Nonanal	1100	1389	-	0.72 ± 0.01	-	--	-	0.72 ± 0.05
(*E*)*-β-*Damascenone	1384	1819	4.82 ± 0.01	5.55 ± 0.01	-	0.86 ± 0.01	1.05 ± 0.1	5.34 ± 0.01
*β*-Ionone	1487	1935	2.29 ± 0.01	13.13 ± 0.01	6.01 ± 0.01	3.75 ± 0.01	6.83 ± 0.01	11.33 ± 0.01
Hexadecanoic acid *	1959	2912	5.61 ± 0.01	8.25 ± 0.01	27.66 ± 0.01	17.86 ± 0.01	17.73 ± 0.01	12.57 ± 0.01
Hydrocarbons			11.2	17.11	23.02	21.13	4.04	28.16
Heneicosane *	2100	2100	0.81 ± 0.05	2.21 ± 0.01	-	0.45 ± 0.02	0.30 ± 0.01	-
Docosane *	2200	2200	0.28 ± 0.01	1.47 ± 0.01	0.32 ± 0.01	1.42 ± 0.01	0.13 ± 0.01	1.92 ± 0.01
Tricosane *	2300	2300	0.25 ± 0.07	3.42 ± 0.01	9.11 ± 0.01	0.65 ± 0.02	0.15 ± 0.01	0.53 ± 0.01
Tetracosane *	2400	2400	8.48 ± 0.01	1.17 ± 0.01	5.63 ± 0.01	0.57 ± 0.01	0.26 ± 0.01	0.56 ± 0.01
Pentacosane *	2500	2500	-	6.75 ± 0.01	2.27 ± 0.01	0.36 ± 0.01	0.28 ± 0.01	0.21 ± 0.01
Hexacosane *	2600	2600	-	0.65 ± 0.01	0.17 ± 0.01	0.33 ± 0.05	0.46 ± 0.01	6.57 ± 0.01
Heptacosane *	2700	2700	-	0.32 ± 0.01	0.32 ± 0.05	11.82 ± 0.01	0.53 ± 0.01	17.21 ± 0.01
Octacosane *	2800	2800	1.38 ± 0.01	1.12 ± 0.1	5.2 ± 0.01	5.53 ± 0.01	1.93 ± 0.01	1.16 ± 0.01
Total identification (%)			95.79	95.44	95.86	94.87	95.64	96.8

Retention indices (RIs) were determined relative to a series of n-alkanes (C8–C40) on capillary columns VF5-ms (RI^a^) and CPWax 52 (RI^b^); identification method: RI, comparison of RIs with those in a self-generated library reported in the literature [27] and/or with authentic samples; comparison of mass spectra with those in the NIST02 and Wiley 9 mass spectral libraries; * co-injection with reference compounds; -, not identified; SD, standard deviation of triplicate analysis.

**Table 3:** Constituents of the hydrosols (HYs, %) obtained by microwave extraction of six *Veronica* species.

			*V. agrestis*	*V. anagalloides*	*V. austriaca* ssp. *jacquini*i	*V. beccabunga*	*V. cymbalaria*	*V. officinalis*
Component	RI^a^	RI^b^	HY ± SD	HY ± SD	HY ± SD	HY ± SD	HY ± SD	HY ± SD
Monoterpene hydrocarbons			6.38	3.81	-	17.11	1.28	0.54
*α*-Thujene	924	1012	4.32 ± 0.01	-	-	-	-	-
*α*-Pinene *	935	1017	2.06 ± 0.01	3.81 ± 0.01	-	17.11 ± 0.01	1.28 ± 0.01	0.54 ± 0.01
Oxygenated monoterpenes			10.16	6.37	8.2	23.85	2.18	13.1
*γ*-Terpinene	1057	1225	-	-	0.53 ± 0.01	-	-	-
Linalool	1095	1506	5.83 ± 0.01	4.27 ± 0.01	-	3.11 ± 0.01	1.73 ± 0.01	6.61 ± 0.01
Terpinen-4-*ol*	1174	1686	0.34 ± 0.01	1.45 ± 0.01	0.66 ± 0.01	0.34 ± 0.01	0.45 ± 0.01	6.49 ± 0.01
*α-*Terpineol	1184	1660	0.72 ± 0.01	0.65 ± 0.01	-	-	-	-
*trans-p-*Mentha-1(7),8-dien-2-ol	1187	1803	0.66 ± 0.01	-	7.01 ± 0.01	0.86 ± 0.01	-	-
Piperitone	1250	1719	2.61 ± 0.01	-	-	19.54 ± 0.01	-	-
Sesquiterpene hydrocarbons			9.77	8.86	12.07	7.73	2.45	2.15
(*E*)*-*Caryophyllene *	1424	1585	5.37 ± 0.01	4.32 ± 0.01	6.31 ± 0.01	6.21 ± 0.01	0.97 ± 0.07	1.44 ± 0.01
*allo-*Aromadendrene	1465	1662	1.01 ± 0.01	2.21 ± 0.05	0.75 ± 0.03	0.67 ± 0.01	0.41 ± 0.01	0.71 ± 0.01
Germacrene D	1481	1692	2.34 ± 0.01	0.80 ± 0.01	2.15 ± 0.01	0.85 ± 0.01	0.26 ± 0.01	-
*δ-*Selinene	1492	1756	0.73 ± 0.01	1.53 ± 0.01	2.86 ± 0.1	-	0.81 ± 0.01	-
*δ*-Cadinene	1517	1745	0.32 ± 0.01	-	-	-	-	-
Oxygenated sesquiterpenes			17.34	7.98	9.46	5.08	7.58	3.99
Spathulenol	1577	2101	0.36 ± 0.1	-	0.46 ± 0.01	-	0.55 ± 0.01	-
Caryophyllene oxide *	1581	1955	14.01 ± 0.01	5.15 ± 0.01	5.35 ± 0.01	2.19 ± 0.01	6.26 ± 0.01	1.42 ± 0.1
Viridiflorol	1592	2099	0.85 ± 0.01	0.43 ± 0.01	0.86 ± 0.03	1.18 ± 0.01	-	-
*γ-*Eudesmol	1632	2175	2.12 ± 0.05	1.65 ± 0.01	-	0.69 ± 0.01	-	-
*α*-Muurolol	1645	2181	-	-	1.77 ± 0.01	0.44 ± 0.07	0.65 ± 0.01	1.64 ± 0.01
*α*-Bisabolol	1685	2210	-	-	0.45 ± 0.01	0.58 ± 0.01	0.12 ± 0.01	0.61 ± 0.01
*α-*Bisabolol oxide	1748	2511	-	0.75 ± 0.01	-	-	-	0.32 ± 0.01
Hexahydrofarnesyl acetone *	1839	2113	-	-	0.57 ± 0.01	-	-	-
Phenolic compounds			11.37	13.57	42.58	1.15	69.93	30.92
Thymol *	1289	2154	2.32 ± 0.1	-	5.57 ± 0.01	1.15 ± 0.01	-	1.81 ± 0.03
*p*-Vinyl guaicol	1313	2156	-	-	-	-	-	-
Methyl eugenol	1403	2005	9.05 ± 0.01	13.57 ± 0.01	37.01 ± 0.01	-	38.61 ± 0.01	22.01 ± 0.01
(*Z*)-Methyl isoeugenol	1451	2070	-	-	-	-	31.32 ± 0.01	7.12 ± 0.01
Acids, alcohols and esters			42.94	55.13	23.55	38.8	12.08	44.24
Isopentyl acetate	863	1127	-	-	0.22 ± 0.01	-	-	-
Benzaldehyde	952	1508	6.29 ± 0.01	13.56 ± 0.01	6.33 ± 0.01	4.57 ± 0.01	0.13 ± 0.03	10.36 ± 0.01
Benzene acetaldehyde	1036	1633	11.56 ± 0.01	9.67 ± 0.01	13.34 ± 0.01	10.73 ± 0.01	10.81 ± 0.01	3.77 ± 0.03
*n-*Nonanal	1100	1389	2.35 ± 0.1	3.82 ± 0.01	1.57 ± 0.01	-	-	1.26 ± 0.01
(*E*)*-β-*Damascenone	1384	1819	12.42 ± 0.01	11.55 ± 0.01	1.01 ± 0.01	8.32 ± 0.01	-	14.01 ± 0.01
*β*-Ionone	1487	1935	10.32 ± 0.01	16.53 ± 0.01	1.08 ± 0.01	15.18 ± 0.01	1.14 ± 0.01	14.84 ± 0.01
Total identification (%)			97.96	95.72	95.86	93.72	95.5	94.94

Retention indices (RIs) were determined relative to a series of n-alkanes (C8–C40) on capillary columns VF5-ms (RI^a^) and CPWax 52 (RI^b^); identification method: RI, comparison of RIs with those in a self-generated library reported in the literature [27] and/or with authentic samples; comparison of mass spectra with those in the NIST02 and Wiley 9 mass spectral libraries; * injection with reference compounds; -, not identified; SD, standard deviation of triplicate analysis.

## Data Availability

The samples and any additional research data are available from the authors on request.

## Supplementary Materials

The following supporting information can be downloaded at https://www.mdpi.com/article/10.3390/plants12183244/s1, Figure S1: Antiproliferative activity of *V. agrestis* EO on MDA-MB-231 cancer cell line; Figure S2. Antiproliferative activity of *V. agrestis* EO on T24 cancer cell line; Figure S3. Antiproliferative activity of *V. agrestis* HY on MDA-MB-231 cancer cell line; Figure S4. Antiproliferative activity of *V. agrestis* HY on T24 cancer cell line; Figure S5. Antiproliferative activity of *V. anagalloides* EO on MDA-MB-231 cancer cell line; Figure S6. Antiproliferative activity of *V. anagalloides* EO on T24 cancer cell line; Figure S7. Antiproliferative activity of *V. anagalloides* HY on MDA-MB-231 cancer cell lines; Figure S8. Antiproliferative activity of *V. anagalloides* HY on T24 cancer cell line; Figure S9. Antiproliferative activity of *V. austriaca* ssp. *jacquini* EO on MDA-MB-231 cancer cell line; Figure S10. Antiproliferative activity of *V. austriaca* ssp. *jacquini* EO on T24 cancer cell line; Figure S11. Antiproliferative activity of *V. austriaca* ssp. *jacquini* HY on MDA-MB-231 cancer cell line; Figure S12. Antiproliferative activity of *V. austriaca* ssp. *jacquini* HY on T24 cancer cell line; Figure S13. Antiproliferative activity of *V. beccabunga* EO on MDA-MB-231 cancer cell line; Figure S14. Antiproliferative activity of *V. beccabunga* EO on T24 cancer cell line; Figure S15. Antiproliferative activity of *V. beccabunga* HY on MDA-MB-231 cancer cell line; Figure S16. Antiproliferative activity of *V. beccabunga* HY on T24 cancer cell line; Figure S17. Antiproliferative activity of *V. cymbalaria* EO on MDA-MB-231 cancer cell line; Figure S18. Antiproliferative activity of *V. cymbalaria* EO on T24 cancer cell line; Figure S19. Antiproliferative activity of *V. cymbalaria* HY on MDA-MB-231 cancer cell line; Figure S20. Antiproliferative activity of *V. cymbalaria* HY on T24 cancer cell line; Figure S21. Antiproliferative activity of *V. officinalis* EO on MDA-MB-231 cancer cell line; Figure S22. Antiproliferative activity of *V. officinalis* EO on T24 cancer cell line; Figure S23. Antiproliferative activity of *V. officinalis* HY on MDA-MB-231 cancer cell line; Figure S24. Antiproliferative activity of *V. officinalis* HY on T24 cancer cell line.
