# Supplementary figures and images for: Isolation of Volatile Compounds by Microwave-Assisted Extraction from Six Veronica Species and Testing of Their Antiproliferative and Apoptotic Activities
Source: Plants (Basel). 2023 Sep 12;12(18):3244. doi: 10.3390/plants12183244 (PMC10535125; doi:10.3390/plants12183244)

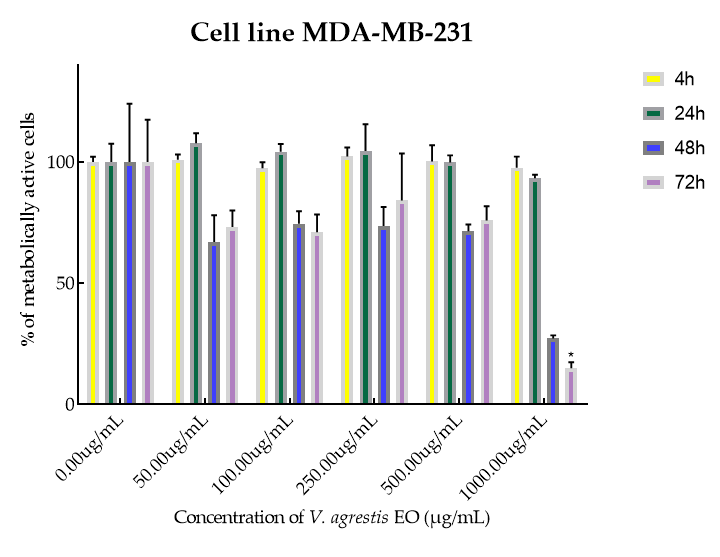

Supplement: Supplementary file 1 [file plants-12-03244-s001.zip › Figure S1. Antiproliferative activity of V. agrestis EO on MDA-MB-231 cancer cell line.tif]

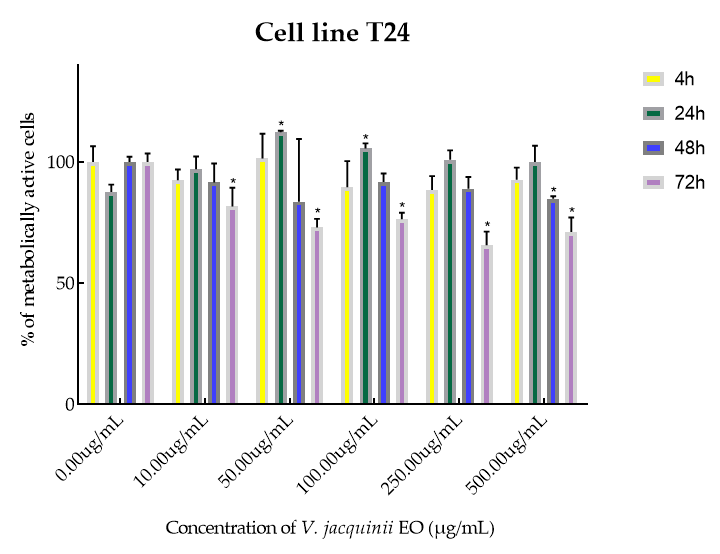

Supplement: Supplementary file 1 [file plants-12-03244-s001.zip › Figure S10. Antiproliferative activity of V. austriaca ssp. jacquini EO on T24 cancer cell line.tif]

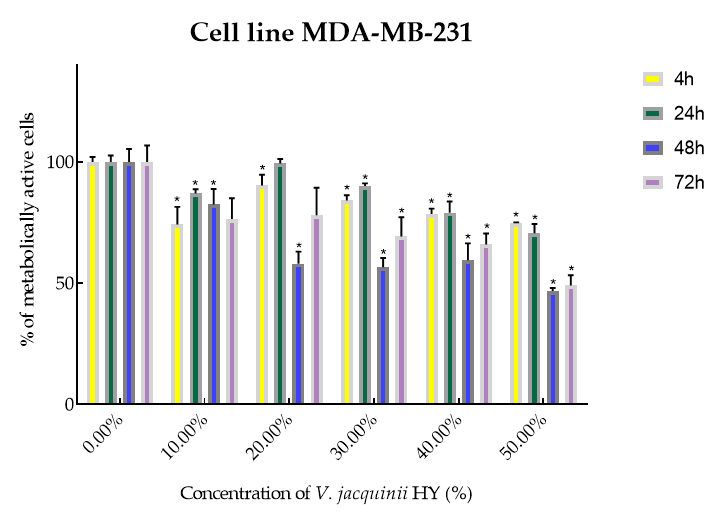

Supplement: Supplementary file 1 [file plants-12-03244-s001.zip › Figure S11. Antiproliferative activity of V. austriaca ssp. jacquini HY on MDA-MB-231 cancer cell line.tif]

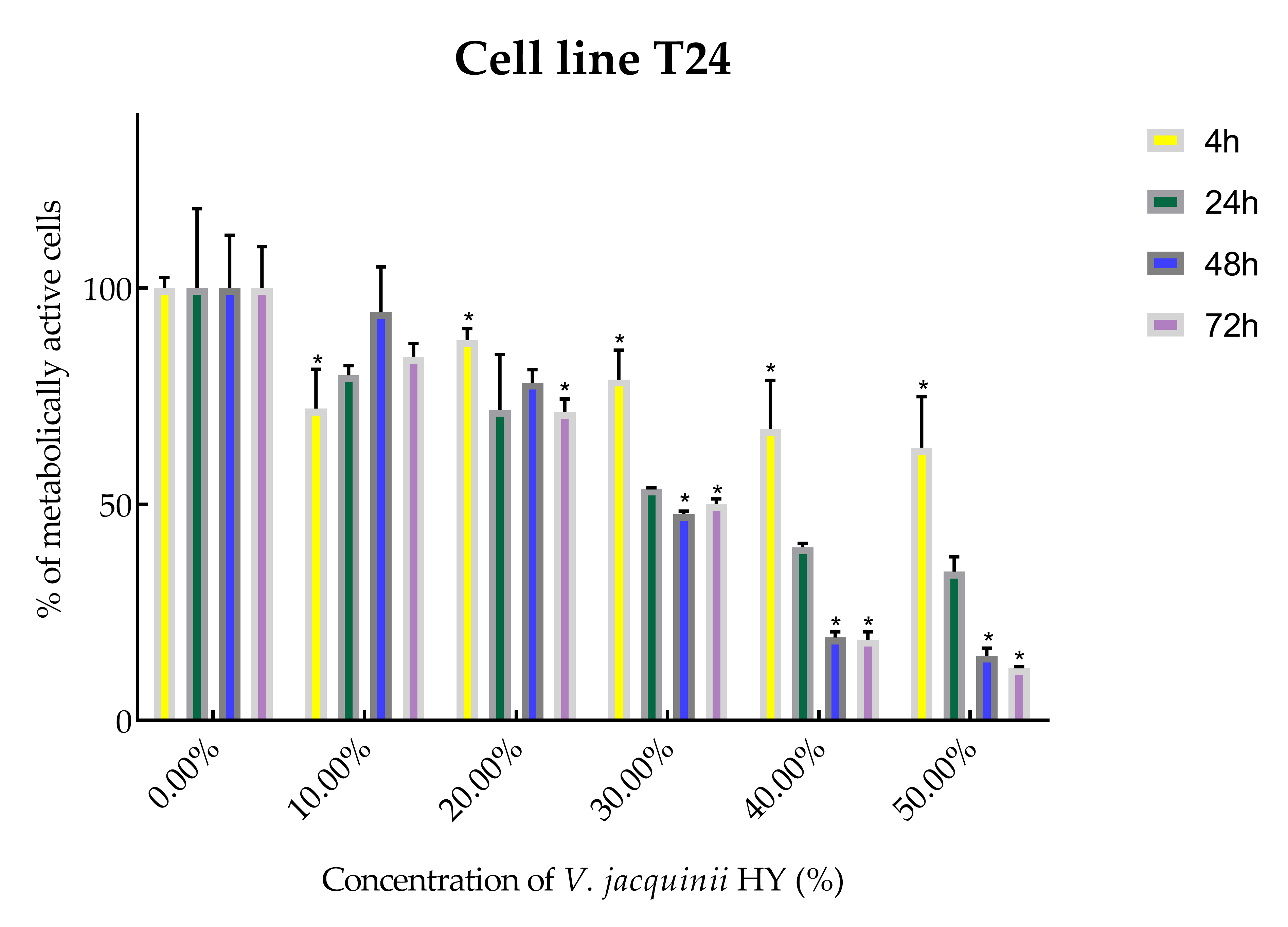

Supplement: Supplementary file 1 [file plants-12-03244-s001.zip › Figure S12. Antiproliferative activity of V. austriaca ssp. jacquini HY on T24 cancer cell line.tif]

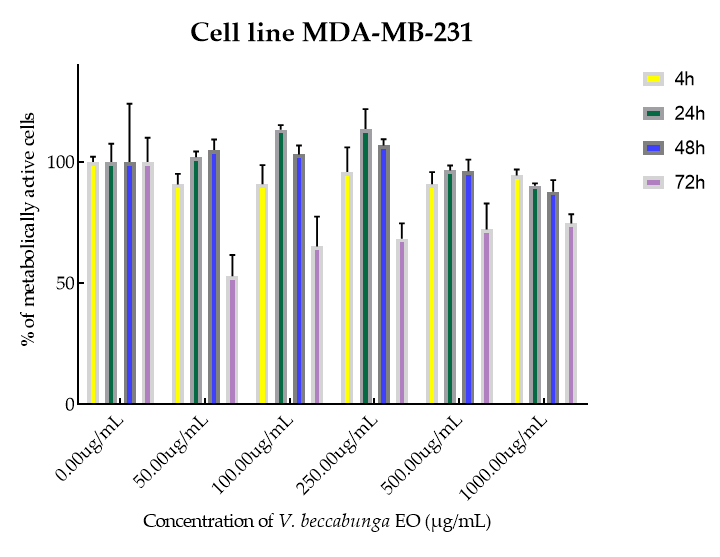

Supplement: Supplementary file 1 [file plants-12-03244-s001.zip › Figure S13. Antiproliferative activity of V. beccabunga EO on MDA-MB-231 cancer cell line.tif]

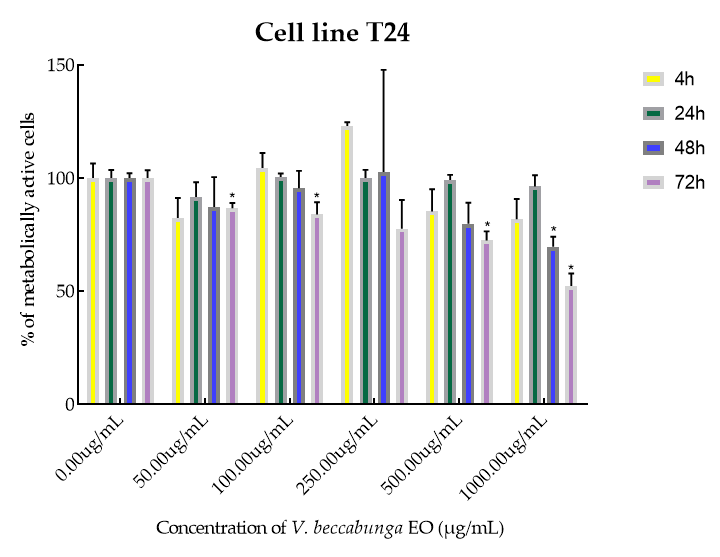

Supplement: Supplementary file 1 [file plants-12-03244-s001.zip › Figure S14. Antiproliferative activity of V. beccabunga EO on T24 cancer cell line.tif]

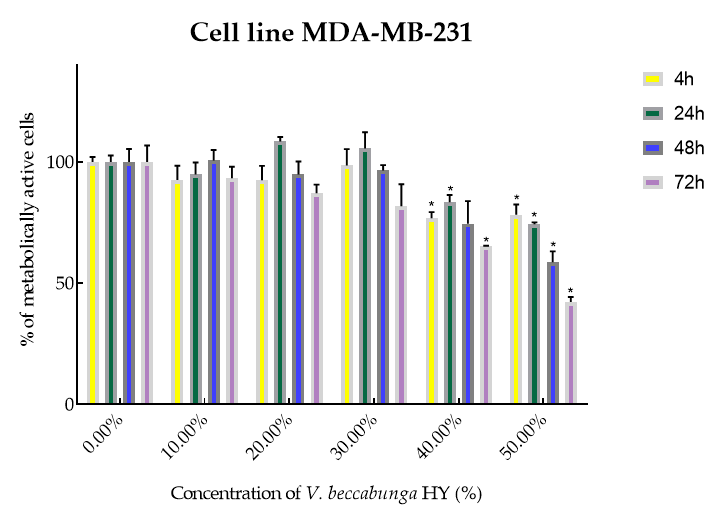

Supplement: Supplementary file 1 [file plants-12-03244-s001.zip › Figure S15. Antiproliferative activity of V. beccabunga HY on MDA-MB-231 cancer cell line.tif]

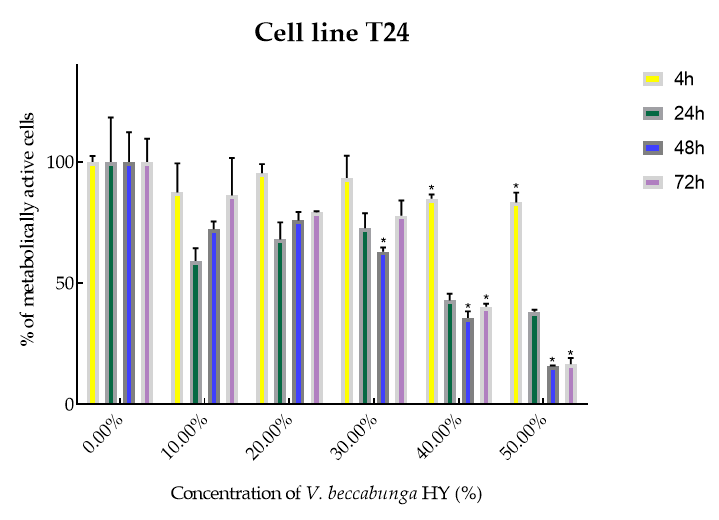

Supplement: Supplementary file 1 [file plants-12-03244-s001.zip › Figure S16. Antiproliferative activity of V. beccabunga HY on T24 cancer cell line.tif]

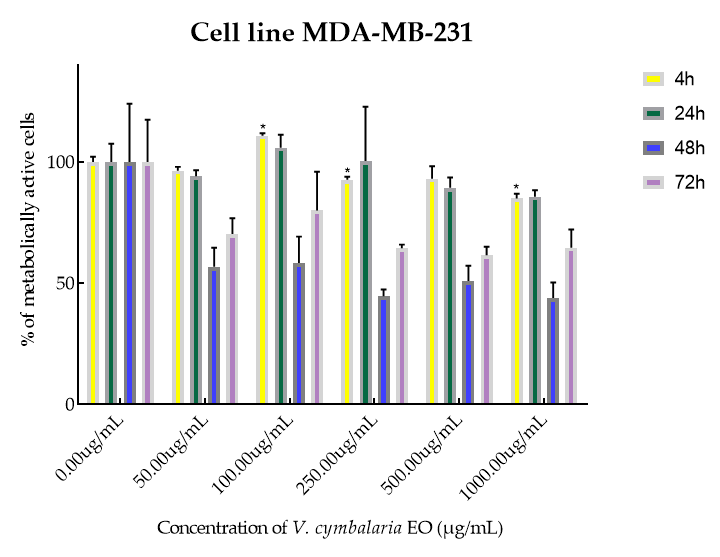

Supplement: Supplementary file 1 [file plants-12-03244-s001.zip › Figure S17. Antiproliferative activity of V. cymbalaria EO on MDA-MB-231 cancer cell line.tif]

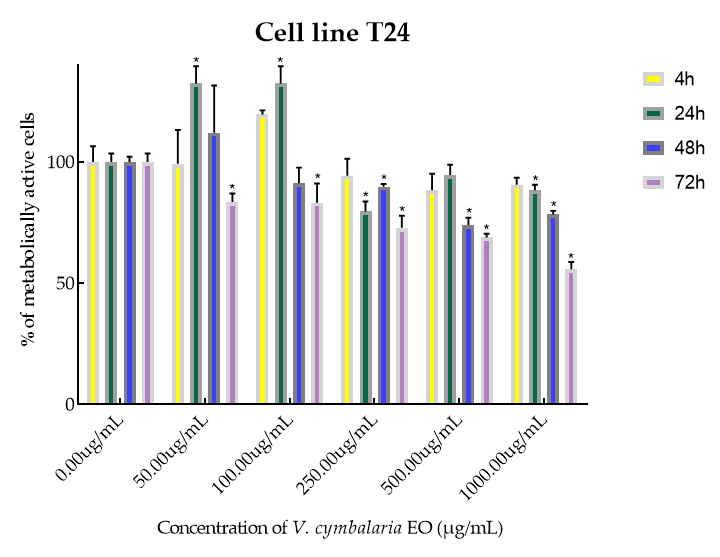

Supplement: Supplementary file 1 [file plants-12-03244-s001.zip › Figure S18. Antiproliferative activity of V. cymbalaria EO on T24 cancer cell line.tif]

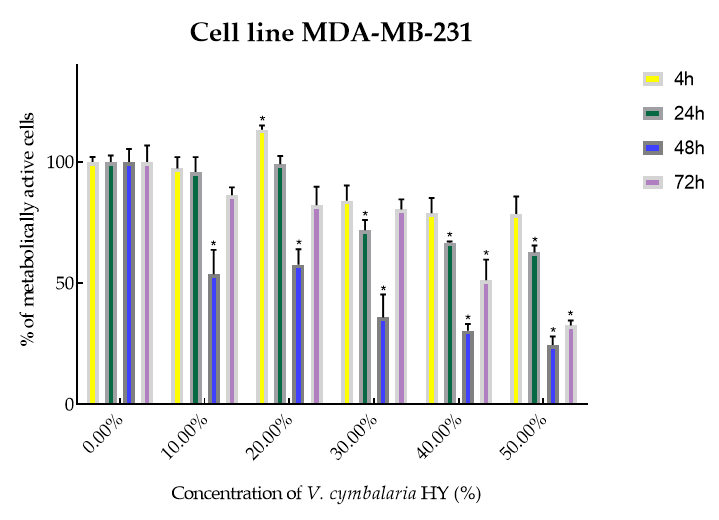

Supplement: Supplementary file 1 [file plants-12-03244-s001.zip › Figure S19. Antiproliferative activity of V. cymbalaria HY on MDA-MB-231 cancer cell line.tif]

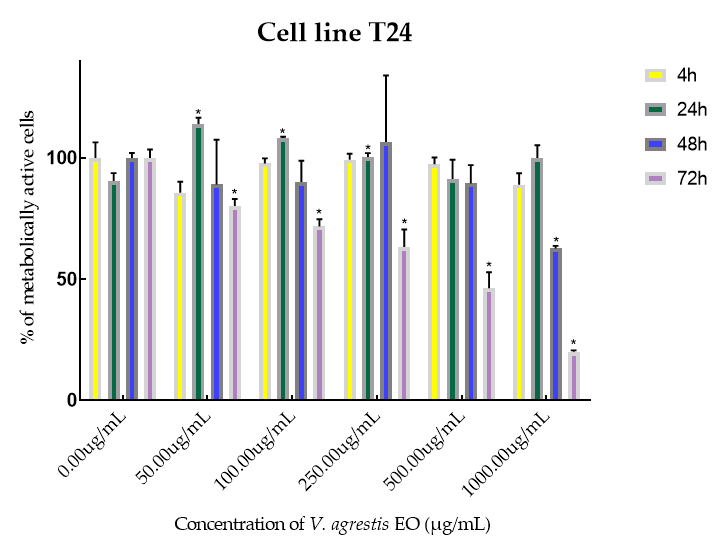

Supplement: Supplementary file 1 [file plants-12-03244-s001.zip › Figure S2. Antiproliferative activity of V. agrestis EO on T24 cancer cell line.tif]

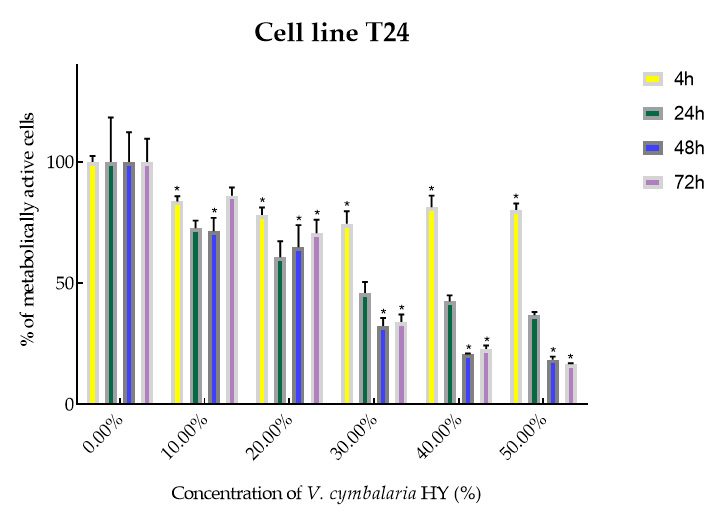

Supplement: Supplementary file 1 [file plants-12-03244-s001.zip › Figure S20. Antiproliferative activity of V. cymbalaria HY on T24 cancer cell line.tif]

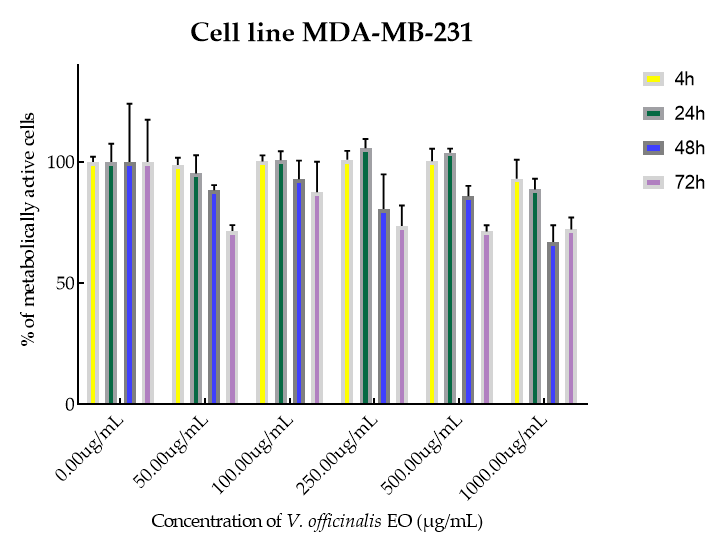

Supplement: Supplementary file 1 [file plants-12-03244-s001.zip › Figure S21. Antiproliferative activity of V. officinalis EO on MDA-MB-231 cancer cell line.tif]

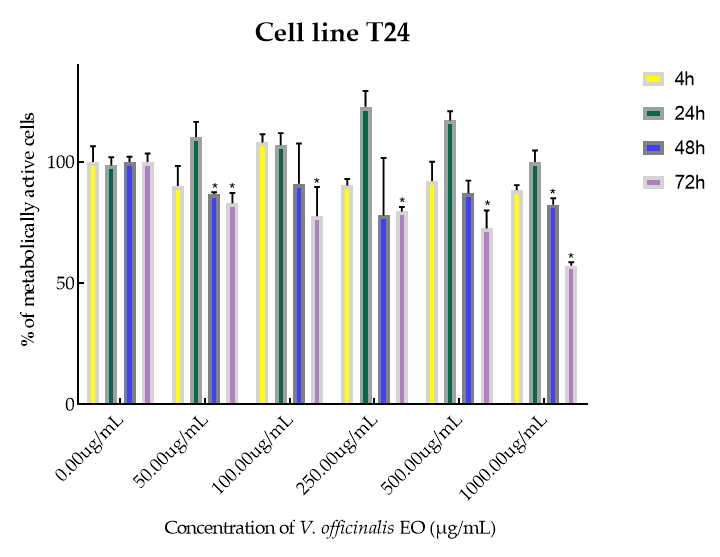

Supplement: Supplementary file 1 [file plants-12-03244-s001.zip › Figure S22. Antiproliferative activity of V. officinalis EO on T24 cancer cell line.tif]

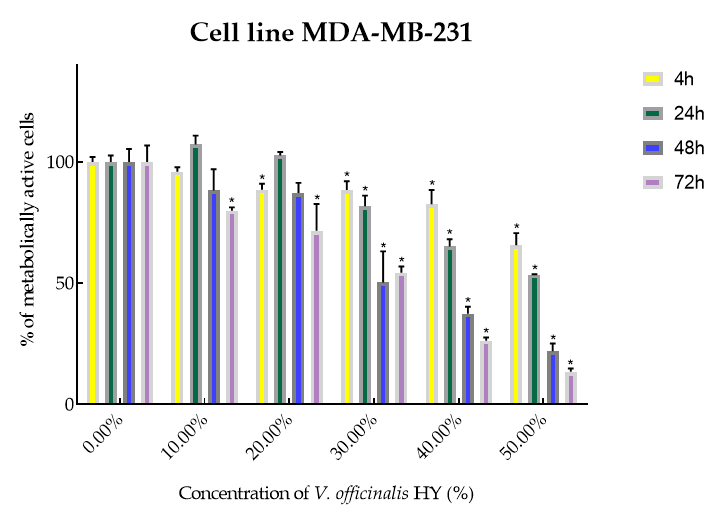

Supplement: Supplementary file 1 [file plants-12-03244-s001.zip › Figure S23. Antiproliferative activity of V. officinalis HY on MDA-MB-231 cancer cell line.tif]

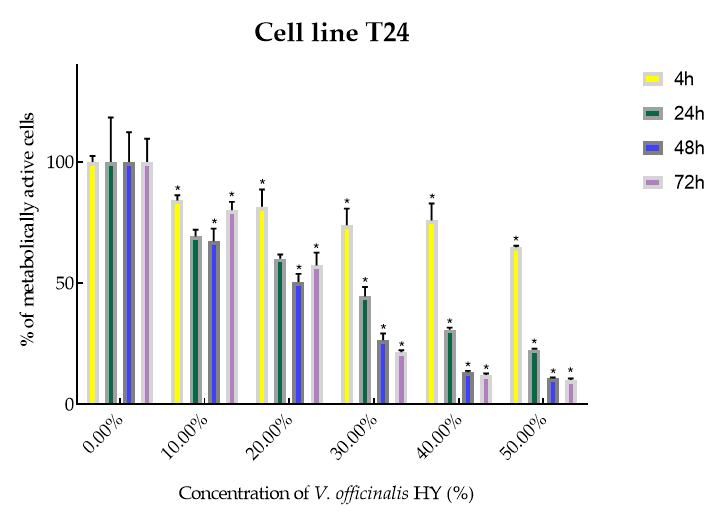

Supplement: Supplementary file 1 [file plants-12-03244-s001.zip › Figure S24. Antiproliferative activity of V. officinalis HY on T24 cancer cell line.tif]

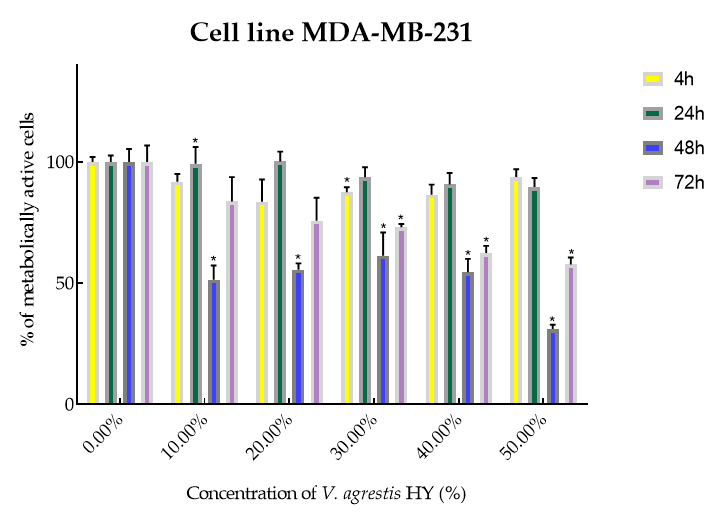

Supplement: Supplementary file 1 [file plants-12-03244-s001.zip › Figure S3. Antiproliferative activity of V. agrestis HY on MDA-MB-231 cancer cell line.tif]

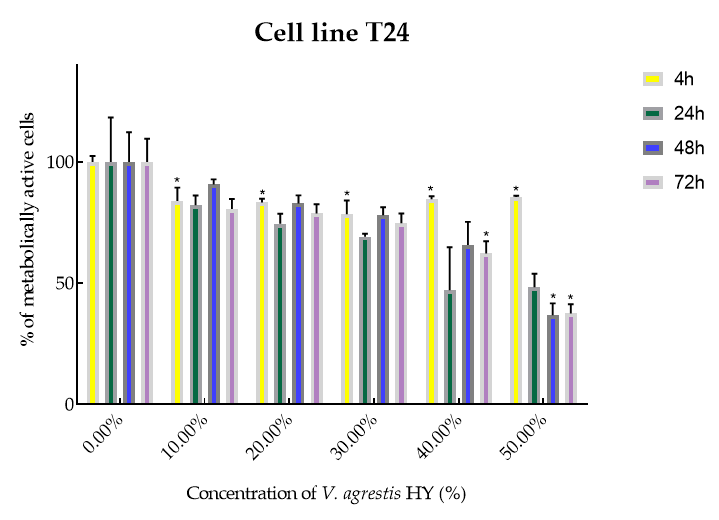

Supplement: Supplementary file 1 [file plants-12-03244-s001.zip › Figure S4. Antiproliferative activity of V. agrestis HY on T24 cancer cell line.tif]

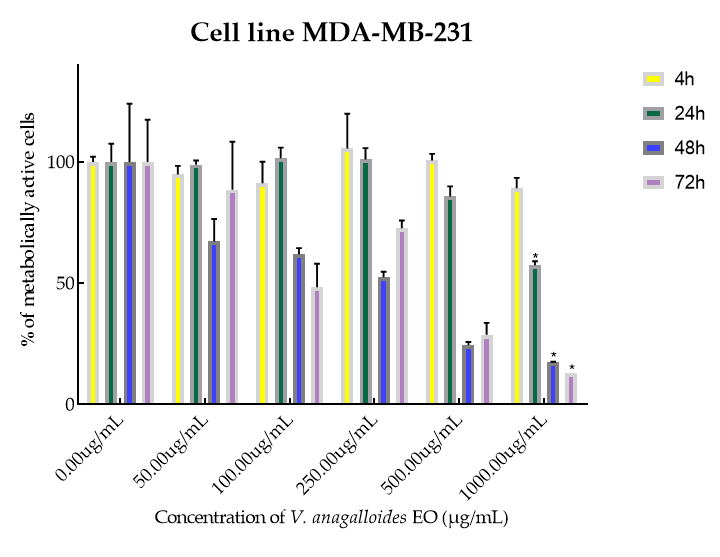

Supplement: Supplementary file 1 [file plants-12-03244-s001.zip › Figure S5. Antiproliferative activity of V. anagalloides EO on MDA-MB-231 cancer cell line.tif]

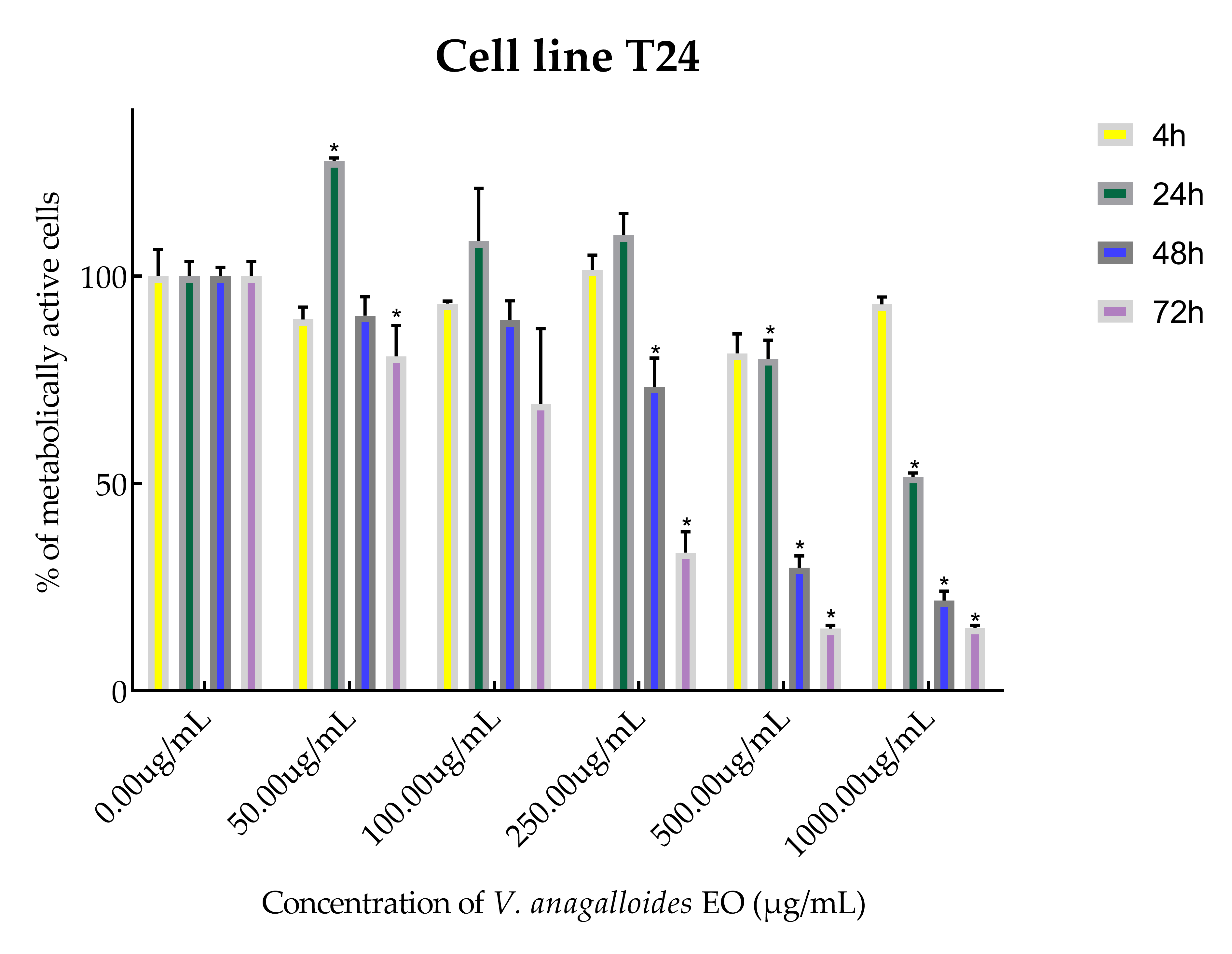

Supplement: Supplementary file 1 [file plants-12-03244-s001.zip › Figure S6. Antiproliferative activity of V. anagalloides EO on T24 cancer cell line.tif]

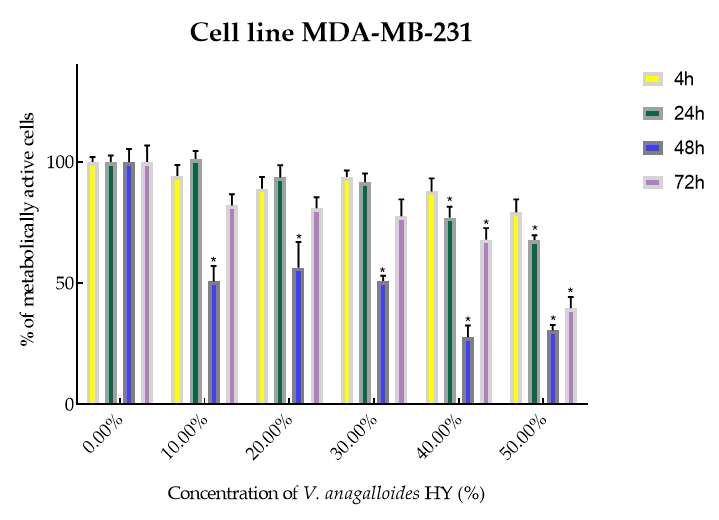

Supplement: Supplementary file 1 [file plants-12-03244-s001.zip › Figure S7. Antiproliferative activity of V. anagalloides HY on MDA-MB-231 cancer cell lines.tif]

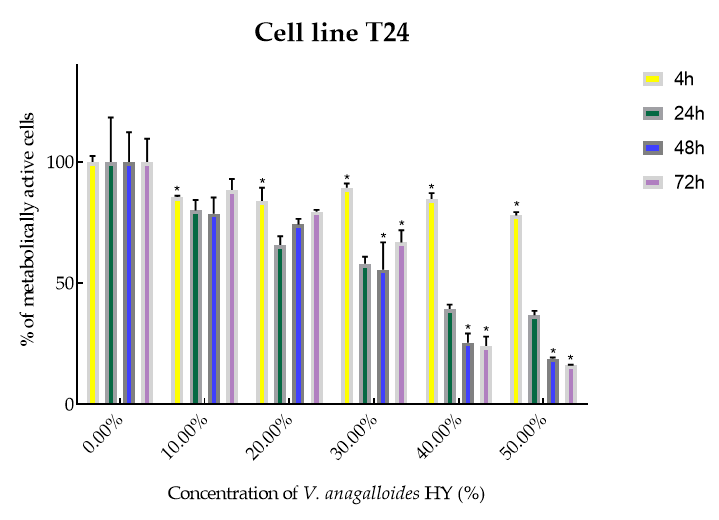

Supplement: Supplementary file 1 [file plants-12-03244-s001.zip › Figure S8. Antiproliferative activity of V. anagalloides HY on T24 cancer cell line.tif]

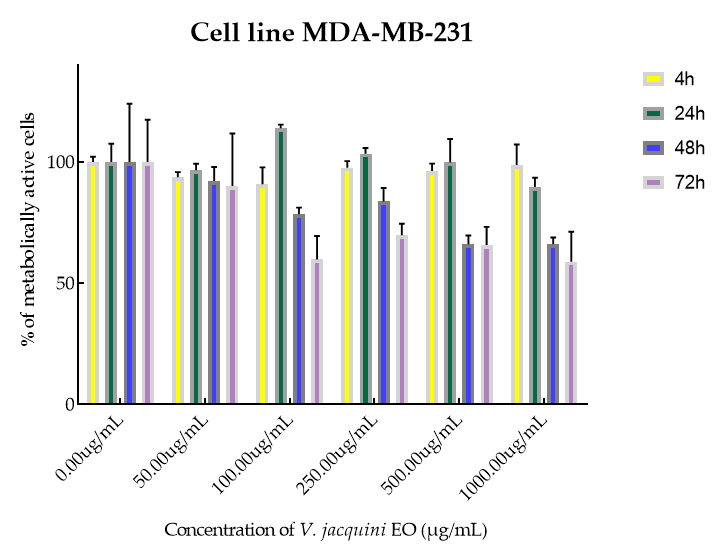

Supplement: Supplementary file 1 [file plants-12-03244-s001.zip › Figure S9. Antiproliferative activity of V. austriaca ssp. jacquini EO on MDA-MB-231 cancer cell line.tif]
